# Synthesis of Linear and Branched Polycarbonate Polyols via Double Metal Cyanide-Catalyzed Ring-Opening (Co)polymerization of Epoxides

**DOI:** 10.3390/polym17182458

**Published:** 2025-09-11

**Authors:** Won Seok Jae, Ha-Kyung Choi, Han Su Lee, Chinh Hoang Tran, Chi Le Hoang Tran, Khoa Anh Trinh, Il Kim

**Affiliations:** 1 School of Chemical Engineering, Pusan National University, Busandaehag-ro 63-2, Geumjeong-gu, Busan 46241, Republic of Korea; coly2343@pusan.ac.kr (W.S.J.); aomghwm0116@pusan.ac.kr (H.-K.C.); tyghbn22@pusan.ac.kr (H.S.L.); 2 Institute of Advanced Technology, Vietnam Academy of Science and Technology, TL29 Street, An Phu Dong, Ho Chi Minh 70000, Vietnam; 3 FPT University, FPT Polytechnic, Nguyen Van Cu Street, An Binh, Can Tho 90000, Vietnam; chithl@fpt.edu.vn (C.L.H.T.); khoata17@fe.edu.vn (K.A.T.)

**Keywords:** double metal cyanide catalyst, epoxides, ring-opening polymerization, carbon dioxide, polyol

## Abstract

A series of polyether and poly(ether carbonate) polyols have been synthesized via Zn(II)-Co(III) double metal cyanide (DMC)-catalyzed ring-opening (co)polymerization of various epoxides, such as propylene oxide, 1,2-epoxybutane, epichlorohydrin, styrene oxide, and glycidol, with and without CO_2_. The resulting polyether polyols exhibit linear and branched architectures (degrees of branching, DB = 0.27), high catalytic activities with turnover frequencies up to 461 min^−1^, narrow dispersities (1.15–1.25), and low levels of unsaturation (0.004 meq g^−1^). The DMC catalysts also enable the efficient synthesis of poly(propylene carbonate) polyol with carbonate contents up to 40% and yields reaching 63%. Additionally, branched poly(ether carbonate) polyols with tunable DB values (0.14–0.21), yields up to 70%, and carbonate contents up to 33% are synthesized via CO_2_ fixation to glycidol. The synthesized polyols hold strong potential for industrial applications in polyurethanes and other advanced materials, offering versatile performance for use in coatings, adhesives, sealants, and elastomers. Overall, this study highlights the effectiveness of DMC catalysts in producing high-performance polyols, contributing to the development of sustainable materials with precise architectural control.

## 1. Introduction

Global warming has emerged as a critical environmental concern worldwide over the past several decades, primarily driven by the release of greenhouse gas emissions, especially carbon dioxide (CO_2_), into the atmosphere [1]. Consequently, numerous countries and institutions are actively engaged in efforts to mitigate CO_2_ emissions. Among the various strategies, carbon capture, utilization, and storage (CCUS) technologies are recognized as one of the effective approaches for reducing global CO_2_ levels [2] into commercially valuable chemicals, including the *N*-formylation of amines with CO_2_ [3], hydrogenation of CO_2_ [4,5], and the fixation of CO_2_ to epoxides [6,7,8]. The latter method, CO_2_ fixation to epoxides, holds considerable industrial promise within CCUS, particularly for the production of polyurethanes [9]. The pioneering work in this field was reported by Inoue et al. in 1969, describing the copolymerization of propylene oxide (PO) and CO_2_ using a ZnEt_2_ catalyst [10]. Since then, extensive studies have investigated a wide range of catalytic systems, including Cr^III^(salen)Cl, cobalt–salen complexes with sulfonate anions, zinc glutarate, iron-based binary catalysts, and zinc gallate [11,12,13,14,15,16,17,18,19]. Despite their effectiveness, homogeneous catalytic systems present several challenges, such as residual toxic species in the final product, formation of monofunctional fractions, complex synthesis routes, difficulties in separating catalysts from products, and limited recyclability, all of which restrict their industrial applicability [20,21]. In contrast, heterogeneous catalysts offer clear advantages in terms of separation and reusability. However, they generally yield mixtures of cyclic carbonates and poly(propylene carbonate) (PPC) with lower carbonate linkage content. While homogeneous catalysts typically exhibit superior activity and selectivity in CO_2_/PO copolymerization, producing alternating PPCs in high yields with high carbonate incorporation within short reaction times, the industrial promise of heterogeneous systems lies in their operational simplicity and recyclability, despite current limitations [22].

Double metal cyanide (DMC) catalysts, often referred to as Prussian blue analogs, are well-established as efficient heterogeneous catalysts for ring-opening polymerization (ROP) and ring-opening copolymerization (ROCOP) of epoxide with CO_2_ [23,24,25]. Their structure typically comprises two distinct metal atoms linked by cyanide ligands, an electron-donating complexing agent (CA), and a polyvalent alcohol serving as a co-complexing agent (co-CA). The initial development of DMC catalysts, pioneered by the General Tire and Rubber Company in 1966, utilized diglyme as an organic CA and demonstrated significant catalytic activity in the ring-opening polymerization (ROP) of epoxides [26]. Subsequent research confirmed the critical role of CA molecules in achieving highly active catalysts. Beyond ROP and ROCOP, DMC catalysts have shown considerable potential across a broad spectrum of chemical reactions, including the synthesis of hyperbranched polymers [27,28], lactone polymerization [29,30,31], esterification and transesterification [32,33,34], hydroamination reactions [35,36,37], Prins condensation [38] and oxidation reactions [39]. Conventionally, DMC catalysts are synthesized by precipitating metal salts such as ZnCl_2_ with metal cyanide salts like K_3_Co(CN)_6_, followed by coordination with electron-donating CAs and co-CAs. However, this conventional approach presents several drawbacks, including the need for a large excess of heavy metal salts ([Zn]/[Co] ≈ 6–10), the use of small-molecule organic CAs classified as volatile organic compounds, and repeated CA additions to achieve complete complexation [40]. The resulting catalysts are typically amorphous with poorly defined structures, raising both environmental concerns and challenges for catalyst characterization. To eliminate the use of organic CAs, ball-milling strategies have been developed; however, these methods are associated with relatively high processing costs. To obtain well-defined DMC compounds and improve their activity and selectivity in the ROCOP of PO with CO_2_, several alternative strategies have been explored. For instance, reacting ZnCl_2_ with K_3_Co(CN)_6_ in the presence of acetate ions yielded highly crystalline DMC catalysts with a pure monoclinic structure [41]. Substituting K_3_Co(CN)_6_ with H_3_Co(CN)_6_, combined with post-treatment using PhOLi, HCl, or LiOAc, produced thin hexagonal plate- and straight plate-like DMC compounds [42,43,44]. Similarly, employing tetracyanonickelate in place of hexacyanocobaltate enabled the synthesis of two-dimensional layered DMC catalysts [45]. Hydrothermal synthesis has emerged as a versatile alternative, widely applied in the preparation of dual-metal systems, zeolites, photocatalysts, and carbon-based materials [46,47,48,49,50,51]. This method utilizes hot, pressurized water within a catalyst slurry to induce porosity and enhance catalytic activity, while offering a simplified, one-step route compared to conventional precipitation–complexation methods [52].

In this study, DMC catalysts synthesized via hydrothermal methods were utilized for the polymerization of various epoxides, including PO, 1,2-epoxybutane (EB), styrene oxide (SO), epichlorohydrin (ECH), and glycidol (G). Additionally, the copolymerization of CO_2_ with glycidol was explored to produce multibranched polycarbonate polyols. The chemical structures and quality of the resulting polyols were characterized using nuclear magnetic resonance (NMR) spectroscopy and gel permeation chromatography (GPC). Further evaluations of the polyols, including unsaturation levels and hydroxyl values, were conducted following ASTM standard procedures.

## 2. Materials and Methods

### 2.1. Materials

Potassium hexacyanocobaltate (III) (K_3_Co(CN)_6_; ≥97%), anhydrous zinc chloride (ZnCl_2_, 98%), hydrochloric acid (37 wt.%), EB (99%), ECH (≥99%), and SO (≥98%) were purchased from Fisher Scientific Korea (Seoul, Republic of Korea) and used without further purification. Glycidol (96%) and poly(ethylene glycol)-*block*-poly(propylene glycol)-*block*-poly(ethylene glycol) (PEG-PPG-PEG) triblock copolymer (MW = 1900 g mol^−1^) were obtained from Sigma-Aldrich (St. Louis, MO, USA) and used as received. Poly(propylene glycol) (PPG, MW = 400 g mol^−1^, functionality = 2) was obtained from Kumho Petrochemical (Ulsan, Republic of Korea). Toluene was purchased from Dae Jung Chemical Co. (Gyeonggi-do, Republic of Korea) and dried over anhydrous magnesium sulfate to remove moisture prior to use. CO_2_ (99.99%) was obtained from Donghae Gas Ind. (Ulsan, Republic of Korea). PO was provided by SK pucore (Ulsan, Republic of Korea).

### 2.2. Preparation of DMC Catalyst

The DMC catalyst was synthesized using ZnCl_2_ as the metal salt and K_3_Co(CN)_6_ as the metal cyanide salt, with a triblock copolymer template under acidic conditions followed by hydrothermal treatment. Briefly, ZnCl_2_ (0.542 g, 4 mmol), the PEG-PPG-PEG template (4 wt%), and HCl (2 mL) were dissolved in 9 mL of distilled water (Solution 1). Separately, K_3_Co(CN)_6_ (0.66 g, 2 mmol) was dissolved in 2.5 mL of distilled water (Solution 2). Solution 1 was then combined with Solution 2 in a Teflon-lined vessel and stirred at 35 °C for 1 h. The reaction mixture was subjected to hydrothermal treatment at 110 °C for 1 h in a stainless-steel autoclave. The resulting precipitate was collected by filtration, thoroughly washed with distilled water, and dried under vacuum to constant weight, yielding the HDMC catalyst.

For comparison, DMC-1 was synthesized under ambient conditions without any organic additives, maintaining a [Zn]/[Co] molar ratio of 2:1. In addition, the benchmark DMC-TBA catalyst was prepared following a previously reported procedure [53].

### 2.3. Semi-Batch ROP of PO

Semi-batch polymerization of PO was performed in a 400 mL autoclave reactor (Parr Instrument Company, Moline, IL, USA) equipped with a liquid mass flowmeter and connected to a laptop for real-time data acquisition. The reactor was charged with 20 g of PPG initiator and 30 mg of DMC catalyst, followed by purging with nitrogen for 30 min. Polymerization was carried out at 115 °C with the initial feeding of 15 g of PO. After a short induction period, additional PO was automatically introduced whenever the internal pressure decreased to 20 kPa, indicating the consumption of the initial PO. Feeding was continued until a total PO consumption of 200 g was reached.

### 2.4. Batch ROP of Epoxides

Batch polymerization of PO and other epoxides were conducted in a 10 mL septum-capped glass autoclave under dry nitrogen using standard Schlenk techniques. A defined amount of PPG initiator and DMC catalyst was added to the reactor, which was purged with nitrogen at 90 °C for 30 min. The required monomer was then added at room temperature, and the reactor was immersed in an oil bath at the desired temperature with vigorous stirring. Upon completion, an aliquot of the crude reaction mixture was withdrawn for analysis. The remaining mixture was dissolved in chloroform, centrifuged to separate the catalyst, and evaporated to obtain the polyol product. The recovered catalyst was treated by Soxhlet extraction with ethanol prior to reuse.

### 2.5. Copolymerization of CO_2_ and Epoxides

Copolymerization was conducted in a 150 mL stainless-steel autoclave (Parr Instrument Company, Moline, IL, USA) designed for high-pressure reactions. In a typical procedure, a predetermined amount of DMC catalyst and PPG initiator were charged into the reactor, which was then purged with CO_2_ for 1 h. After purging, the reactor was cooled to room temperature, and the epoxide (0.3 mol) along with toluene (5–20 mL) was added. The reactor was then pressurized with CO_2_ to 1–3 MPa at room temperature and subsequently heated to 105–140 °C, maintaining these conditions for 3–6 h. Upon completion, the crude reaction mixture was dissolved in chloroform and filtered to remove any residual catalyst. The filtrate was extracted with distilled water to remove cyclic carbonate byproducts and then concentrated under reduced pressure to obtain the polycarbonate polyol.

### 2.6. Characterization

FTIR spectra of the DMC catalysts were recorded over the range of 4000–400 cm^−1^ using the KBr pellet method with a Shimadzu IR Prestige 21 spectrometer (Shimadzu Scientific Korea, Seoul, Republic of Korea). XPS spectra were collected using an ESCALAB 250 X-ray Photoelectron Spectrometer (ThermoFisher Scientific Korea, Seoul, Republic of Korea), equipped with Al Kα radiation (hv = 1486.6 eV), generated by an X-ray source operating at 12 mA and 20 kV XRD patterns were obtained using a Malvern Panalytical X’Pert^3^ X-ray diffractometer (Malvern Panalytical, Great Malvern, UK), scanning over a 2*θ* range of 5–60° at 25 °C with Cu-Kα radiation (λ = 1.541874 Å). Crystallite sizes were estimated from the dominant diffraction peaks using the Debye–Scherrer equation. The catalysts’ microstructures were examined using a Zeiss Gemini 500 field-emission scanning electron microscope (FE-SEM) (Zeiss Korea, Seoul, Republic of Korea), with samples coated with a thin platinum layer to improve conductivity. Pore size distribution and BET surface area measurements were performed using a Micromeritics 3Flex gas adsorption analyzer (Micromeritics, Seoul, Republic of Korea), with samples degassed under vacuum at 100 °C for 12 h and nitrogen as the adsorbate. Catalyst compositions were quantified using ICP-OES on an Optima 8300 instrument (Perkin Elmer, Hopkinton, MA, USA). Elemental analysis (C, H, N, O) was performed using a Vario-Micro Cube CHNS/O analyzer (EA Korea, Hanam, Republic of Korea). Water and polymer template contents were evaluated using thermogravimetric analysis (TGA) on a TGA55 instrument (TA Instruments, Seoul, Republic of Korea) at a heating rate of 10 °C per min from 30 to 800 °C under a nitrogen atmosphere.

^1^H and ^13^C NMR spectra were acquired at 400 MHz using a Varian INOVA 400 NMR spectrometer (Varian Inc., Palo Alto, CA, USA) to analyze the structural characteristics of the polyols. The molecular weights and dispersity (*Ð*) of the polyols were determined by GPC using Waters HSPgel HR columns (6.0 × 150 mm) (Agilent Technologies Inc., Santa Clara, CA, USA), with tetrahydrofuran as the eluent and low *Ð* polystyrene standards for calibration. Hydroxyl values of the polyols were measured using an 88 Titrando titration analyzer with 0.1 N tetrabutylammonium hydroxide (Bu_4_NOH) and *p*-toluenesulfonyl isocyanate (TSI) following ASTM E1899-97 [54]. The degree of unsaturation in the polyether polyols was determined by titration according to ASTM D4671-05 [55].

## 3. Results and Discussion

### 3.1. Characterization of DMC Catalysts

The structural and textural properties of the DMC-1, DMC-TBA, and HDMC catalysts were characterized using various techniques. The FTIR spectra revealed characteristic signals corresponding to the stretching vibrations of −OH and C≡N, and bending vibration of H−O−H and Co−CN at approximately 3400–3500, 2180–2200, 1610, and 449–480 cm^−1^, respectively (Supplementary Figure S1). XPS analysis further confirmed the presence of primary surface elements such as Zn, Co, O, N, and C in these catalysts (Supplementary Figure S2). XRD patterns demonstrated that DMC-1 possessed a highly pure cubic crystalline structure, whereas HDMC displayed a highly crystalline framework comprising both cubic and hexagonal phases (Supplementary Figure S3). In contrast, DMC-TBA exhibited a highly amorphous structure. The chemical compositions of the DMC catalysts were estimated using a combination of ICP-OES, elemental analysis, and TGA (Supplementary Figure S4). Textural analysis revealed that HDMC possessed a significantly higher surface area (354 m^2^ g^−1^) compared to DMC-TBA (245 m^2^ g^−1^) and DMC-1 (40 m^2^ g^−1^), highlighting the advantage of the hydrothermal method in preparing porous and high surface area materials. A summary of the structural and textural properties is provided in Table 1 and Supplementary Table S1.

### 3.2. Synthesis of Polyether Polyols

**Figure 1:**
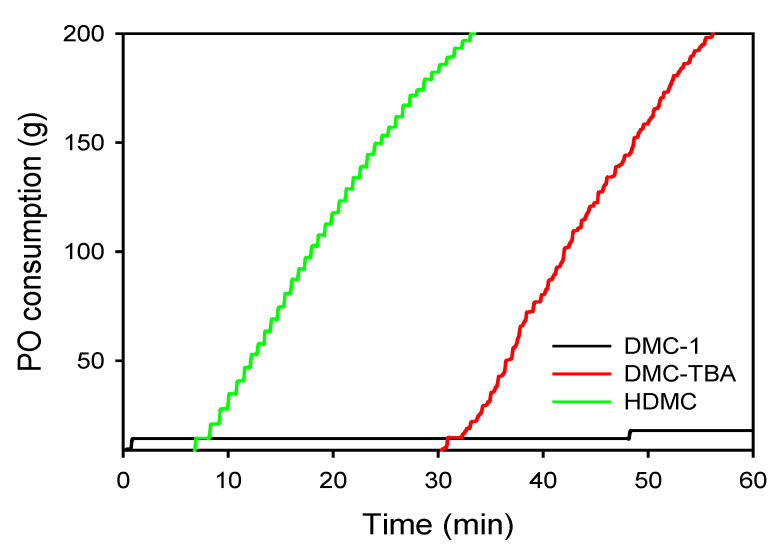
Polymerization rate curves obtained by DMC catalysts. Reaction conditions: PPG = 20 mL, PO = 200 g, DMC catalyst = 0.03–0.1 g, and temperature = 115 °C.

To evaluate catalytic performance, the activity of the HDMC catalyst was investigated in the semi-batch polymerization of PO, with monomer consumption monitored over time. As shown in Figure 1, the HDMC catalyst demonstrated significantly higher activity (TOF = 461 min^−1^) compared to the benchmark DMC-TBA catalyst (TOF = 200 min^−1^), while DMC-1 exhibited negligible activity for the ROP of PO. The superior performance of HDMC can be attributed to its significantly larger surface area, which facilitates greater accessibility of active sites, comparing to DMC-TBA and DMC-1. It is noteworthy that, in addition to Pluronic, we also evaluated a series of DMC catalysts synthesized using polyvinylpyrrolidone (PVP) at varying concentrations as a structure-directing agent. However, none of these catalysts displayed activity in the ROP of PO, despite PVP being commonly employed as a templating agent in PBA synthesis. These findings underscore the critical role of polymeric templates in determining catalyst performance.

**Figure 2:**
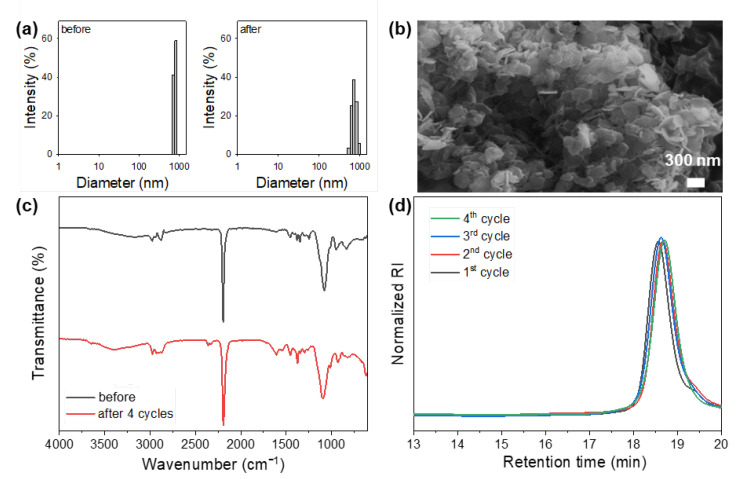
Results of the recycling experiment of HDMC catalyst: (**a**) particle size distributions before and after four cycles. (**b**) SEM image obtained after four cycles. (**c**) FTIR spectra collected before and after four cycles. (**d**) GPC curves of the resultant polyols.

The reusability of the HDMC catalyst was evaluated through the ROP of PO to validate its unique properties in comparison to homogeneous catalysts and highly amorphous DMC catalysts previously reported in the literature. After each cycle, the resulting polyol was dissolved in chloroform, and the catalyst was recovered by centrifugation, followed by ethanol extraction prior to reuse. The DLS analysis revealed no significant change in the particle size distribution of the HDMC catalyst after four consecutive cycles (Figure 2a). Similarly, SEM imaging and FTIR spectra confirmed that both the hexagonal plate-like morphology and the chemical structure of the HDMC catalyst were preserved throughout the recycling experiments (Figure 2b,c). Moreover, the properties of the polyols obtained in each cycle remained consistent (Figure 2d). These findings clearly demonstrate the excellent stability and recyclability of the hydrothermally synthesized HDMC catalyst.

Following this, the HDMC catalyst was employed for the synthesis of linear polyols from various epoxides, including PO, EB, SO, and ECH. As shown in Supplementary Figures S5–S7, the HDMC catalyst afforded high polyol yields (94–99%) across monomers, except for ECH. The slightly higher activity observed for PO ROP is likely due to steric effects related to substituent groups on the monomers. The resulting poly(propylene oxide) polyol exhibited an exceptionally low unsaturation value of 0.004 meq g^−1^, significantly lower than that of the DMC-TBA catalyst (0.007 meq g^−1^), and markedly improved compared to conventional KOH-based catalysts, which typically yield unsaturation levels ranging from 0.01 to 0.04 meq g^−1^. The low degree of unsaturation significantly enhances the thermal and chemical stability of the polyol, making it particularly advantageous for polyurethane production. Such stability improves processing performance and end-use durability, enabling the design of high-quality, functionally tailored polyurethane materials for advanced applications. The MWs of the resulting polyols ranged from 3200 to 6800 g mol^−1^, depending on the epoxide used. All polyols synthesized using a PPG initiator exhibited functionality values between 2.08 and 2.26. Notably, the ^1^H NMR of the crude product from ECH ROP displayed distinct signals corresponding to terminal epoxide groups (Supplementary Figure S8), indicating that the polymer chains were end-capped with epoxy ring. This suggests that ECH polymerization may proceed via a unique initiation mechanism involving chloride ions [56]. As a result, the poly(epichlorohydrin) polyol was obtained with relatively low yield (86%), low MW (1300 g mol^–1^), and poor functionality (0.23), further supporting the hypothesis of an alternative propagation mechanism in the presence of ECH. The results of the ROP of various epoxides are summarized in Table 2.

Branched polyether polyol was synthesized via ring-opening multibranching polymerization (ROMBP) of glycidol using the HDMC catalyst. The latent cyclic AB_2_-type monomer, glycidol, functions both as an initiator and a coordinating ligand, interacting with metal active sites through its hydroxyl and epoxide groups. Polymerization of glycidol yielded branched polyglycidol with a high yield of 91% (Supplementary Figure S9). The ^13^C NMR spectrum displayed characteristic signals corresponding to linear (L_13_, L_14_), dendritic (D), and terminal (T) units, confirming the formation of a branched polyol structure. The number-average degree of polymerization ($\bar{{DP}_{n}}$) and degree of branching (DB) were calculated using Equations (1) and (2), respectively. The core functionality (*f_c_*) of the monomer was assumed to be 2 [57].

(1) $$\bar{{DP}_{n}}=\frac{T+L+D\;}{T-D}f_{c}$$

(2) $$DB=\frac{2D}{2D+L}$$

The resulting polyglycidol exhibited a moderate molecular weight (MW) of 2900 g mol^−1^, a *DB* of 0.27, and an overall functionality of 11.5, consistent with the formation of a semi-branched architecture. Detailed results of the glycidol polymerization are presented in Table 2, run 6.

### 3.3. Synthesis of Poly(ether carbonate) Polyols

Linear and branched poly(ether carbonate) polyols were synthesized via the copolymerization of CO_2_ and epoxides using HDMC catalyst. Reactions were conducted in a stainless-steel high-pressure reactor under a CO_2_ atmosphere (1–3 MPa) at 105–140 °C for 3–6 h. Monomer conversion, product selectivity, chemical structure, and carbonate content of the resulting polyols were analyzed by ^1^H NMR spectroscopy.

**Figure 3:**
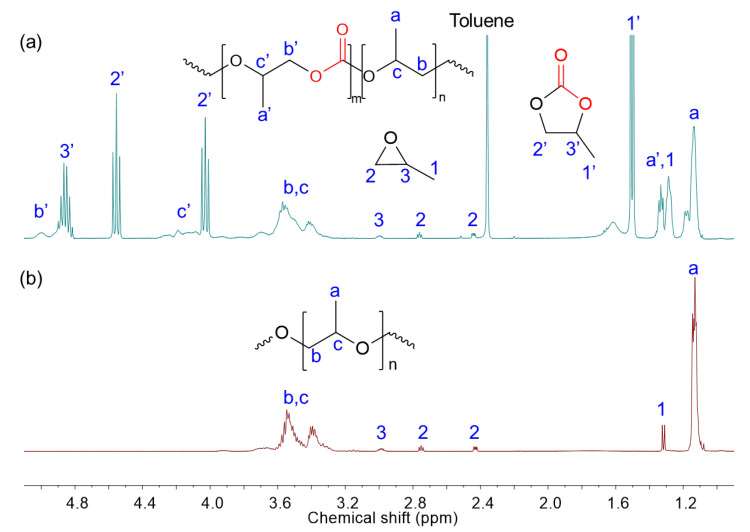
^1^H NMR (400 MHz, CDCl_3_) spectra of the crude reaction mixture of the ROCOP of CO_2_ with PO (**a**) and ROP of PO (**b**) using HDMC catalyst.

For the copolymerization of CO_2_ with PO, signals corresponding to the methyl protons of the carbonate and ether segments appeared at 1.2–1.4 ppm and 1.0–1.2 ppm, respectively. Additional peaks at 4.8, 4.5, 4.0, and 1.5 ppm were attributed to the formation of cyclic carbonate byproducts. Signals in the range of 2.4–3.0 ppm indicated the presence of unreacted PO monomers. The ^1^H NMR spectra of crude reaction mixtures obtained from both the ROP of PO and ROCOP of CO_2_ with PO are shown in Figure 3. Poly(propylene carbonate) (PPC) polyol was obtained with a notable yield of 63.4%, a carbonate content of 40.1%, and a functionality of 2.15. Compared to its polyether counterpart, the resulting PPC polyol exhibited a broader molecular weight distribution (*Đ* = 1.83), likely due to differences in monomer reactivity and propagation kinetics during copolymerization.

**Figure 4:**
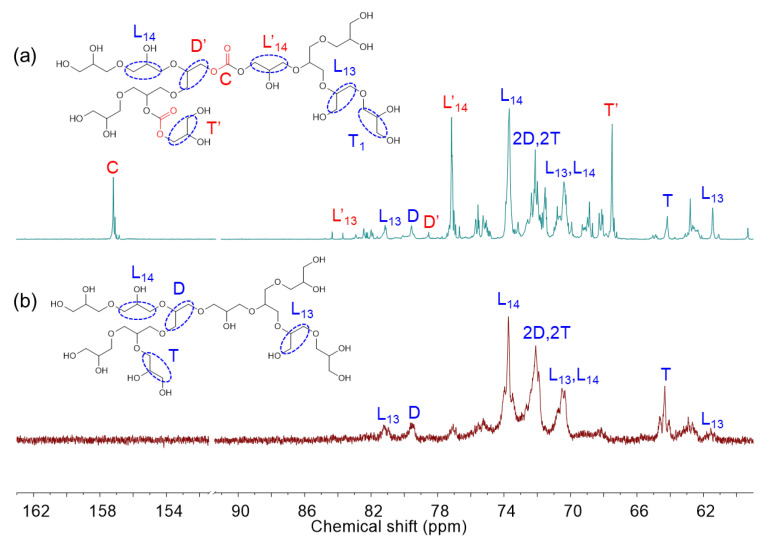
^13^C NMR (400 MHz, CDCl_3_) spectra of the poly(glycidol carbonate) (**a**) and polyglycidol (**b**) using HDMC catalyst. L, T, D, C denote linear, terminal, dendritic, and carbonate components, respectively.

Branched polycarbonate polyols were synthesized via ring-opening multibranching copolymerization of CO_2_ glycidol using HDMC catalysts. The ^1^H and ^13^C NMR spectra of the resulting polyglycidol carbonate (PGC) and the crude reaction mixture are shown in Figure 4 and Supplementary Figure S10 for comparison. In the ^1^H NMR spectra, characteristic signals in the range of 3.4–4.0 ppm and 2.6–3.2 ppm correspond to ether units of the polyol and residual glycidol, respectively. Distinct peaks at 4.9 ppm and 4.8 ppm in the crude mixture are attributed to carbonate units of the polyol and glycidol carbonate byproduct, respectively. Overlapping signals observed at 4.4 and 4.5 ppm are also assigned to carbonate functionalities and glycidol carbonate. In the ^13^C NMR spectra, a prominent signal at 155.4 ppm, absent in the spectrum of polyglycidol, confirms the presence of carbonate (C) species in the copolymer. Additional signals at 76.0 and 66.4 ppm correspond to linear (L′13, and L′14) and terminal (T′) units adjacent to carbonate groups. The $\bar{{DP}_{n}}$ of the PGCs was calculated using Equation (3), and the *DB* was determined using the same approach applied to polyglycidol. The core functionality was set equal to the monomer’s functionality (*f*_c_ = 2).

(3) $$\bar{{DP}_{n}}=\frac{T+L+D+C}{T\;-\;D}f_{c}$$

To evaluate the influence of reaction conditions on the copolymerization of CO_2_ with glycidol, key parameters including temperature, initial CO_2_ pressure, and solvent volume were systematically investigated. As shown in Supplementary Figures S11–S14, reactions performed at temperatures above 120 °C exhibited a clear increase in both glycidol conversion and polyol yield. However, the carbonate content in the resulting polymers decreased under these conditions. This behavior indicated that at elevated temperatures, the propagation of glycidol polymerization is accelerated, favoring the formation of ether linkages over CO_2_ insertion, and CO_2_ solubility in the reaction medium decreases with increasing temperature, thus, reducing the effective concentration of CO_2_ available for incorporation. The combined outcome is enhanced overall productivity at the expense of carbonate selectivity. In contrast, lowering the reaction temperature to 110 °C significantly reduced the carbonate content and the overall monomer conversion. This reduced performance probably arises from insufficient thermal activation of the catalyst, which slows down both epoxide ring-opening and CO_2_ insertion steps. The resulting competition between kinetic limitations at low temperature and CO_2_ mass transfer constraints at higher temperature highlights the importance of optimizing reaction temperature to balance conversion efficiency and carbonate incorporation.

As the initial CO_2_ pressure increased, the total carbonate content rose remarkably from 29.8% to 68.4% (Supplementary Figures S15–S18). However, the carbonate content within the polyol product decreased from 32.7% to 23.1% when the pressure exceeded 2 MPa. Accordingly, polyol yield declined significantly from 70.2% to 29.5%. These observations suggest that higher CO_2_ pressures favor cyclization over polymerization, thereby limiting the incorporation of carbonate units into the polyol backbone.

The influence of solvent (toluene) volume on the reaction was assessed at 120 °C for 6 h, with the CO_2_ pressure maintained at 2 MPa (Supplementary Figures S19–S21). Compared to solvent-free conditions, increasing the volume of toluene enhanced both the carbonate content in the polyol and the total carbonate yield. However, it concurrently led to a reduction in overall conversion and polyol yield. These results suggest that the presence of solvent improves CO_2_ fixation efficiency but promotes cyclization pathways over polymerization. When the solvent volume was increased to 20 mL, both conversion and carbonate content decreased substantially. The solvent may act as a physical diluent or a kinetic inhibitor, further retarding the polymerization process.

The resulting PGCs exhibited *M*_n_ values in the range of 1030–2120 g mol^−1^, lower than that of polyglycidol (2600 g mol^–1^), consistent with the dominance of cyclization over linear growth. The *DB* values of the resulting PGCs ranged from 0.12 to 0.21, aligning with previously reported values for semi-branched polymers. Results for the fixation of CO_2_ to glycidol are summarized in Table 3.

These results clearly demonstrate that HDMC catalyst exhibit strong catalytic activity for CO_2_ fixation. The copolymerization behavior can be tuned by adjusting temperature, pressure, and solvent volume to favor either polymerization or cyclization. Moreover, the resulting cyclic carbonates are easily isolable and offer valuable utility as solvents, electrolytes, or monomeric precursors for advanced polymer materials.

## 4. Conclusions

In this work, we successfully synthesized both linear and branched poly(ether carbonate) polyols through the copolymerization of CO_2_ with various epoxides, using HDMC catalyst. The copolymerization were carried out under mild to moderate pressure and temperature conditions, and the resulting polyols were characterized by comprehensive ^1^H and ^13^C NMR analyses to determine monomer conversion, carbonate content, and polymer microstructure. Linear polyols derived from various epoxides exhibited notable yield and carbonate incorporation, highlighting the effectiveness of the HDMC catalyst in facilitating CO_2_ fixation and ROP. On the other hand, the copolymerization of CO_2_ with glycidol via ROMBP yielded branched PGCs with controlled degrees of branching (*DB* = 0.12–0.21), moderate MWs, and tunable carbonate content.

Reaction parameter studies revealed that elevated temperatures favored polymerization over cyclization, increasing polyol yield but reducing carbonate content. Higher initial CO_2_ pressures promoted cyclization and significantly reduced polyol formation, suggesting that pressure plays a crucial role in dictating reaction pathways. Additionally, solvent effects played a decisive role in governing both conversion and selectivity. The introduction of moderate amounts of toluene improved CO_2_ fixation and favored carbonate incorporation, likely by enhancing monomer solubility and facilitating better mass transfer of CO_2_ into the reaction medium. In contrast, excessive solvent loading reduced the overall conversion, which can be attributed to dilution of active species and decreased local CO_2_ concentration around the catalyst, thereby limiting the effective catalytic turnover.

Overall, our findings confirm that DMC catalysts prepared via a simple hydrothermal method offer a highly tunable and efficient platform for CO_2_-based polymer synthesis. The ability to manipulate reaction parameters allows for precise control over polymer architecture, composition, and branching. Furthermore, the facile recovery and potential utility of the cyclic carbonate byproducts present valuable opportunities for sustainable applications in materials science, such as green solvents, electrolytes, or polymer precursors. This work contributes to the broader goal of utilizing CO_2_ as a renewable carbon feedstock and paves the way for the development of eco-friendly, functional polymer materials.

## Figures and Tables

**Table 1:** Structural and textural properties of the DMC catalysts.

Catalyst	Formula *^a^*	Crystal Structure* ^b^*	*S*_BET_ *^c^* (m^2^ g^−1^)	*V*_p_ *^d^* (cm^3^ g^−1^)	*D*_p_ *^e^* (nm)
DMC-1	Zn_1.40_Co(CN)_5.64_·2.13H_2_O·1.21Cl^−^	cubic	40	0.07	2.8
HDMC	Zn_1.71_Co(CN)_6.53_·0.12PL·1.10H_2_O·1.13Cl^−^	cubic + hexagonal	354	0.14	3.9
DMC-TBA	Zn_2.01_Co(CN)_6.41_·0.53TBA·0.02PL·0.58H_2_O·0.29Cl^−^	amorphous	245	0.10	4.7

*^a^* Estimated by elemental analysis, PL denotes Pluronic template. *^b^* Based on XRD patterns. *^c^* BET surface area. *^d^* Total pore volume. *^e^* Average pore diameter.

**Table 2:** Results for the ROP of epoxides using HDMC catalyst.

Run *^a^*	Monomer	*m*_PPG initiator_ (g)	*t*_p_ (h)	Yield (%)	*M_n_* (g mol^−1^)	*Ð*	$\bar{F}$ *^b^*
1^ *c*^	PO	20	0.5	99.0	6000	1.15	2.15
2	PO	0.2	0.5	99.0	3200	1.15	2.26
3	EB	0.2	0.5	97.8	3400	1.17	2.21
4	SO	0.2	1	94.4	6800	1.25	2.08
5	ECH	0.2	2	86.1	1300	1.16	0.23
6	G	-	6	90.9	2900	1.33	11.5

*^a^* Reaction conditions: HDMC catalyst = 2 mg, monomer = 30 mmol, *T*_P_ = 115 °C. *^b^* Functionality. *^c^* HDMC catalyst = 30 mg, monomer = 3.44 mol.

**Table 3:** Results for the ROCOP of CO_2_ with epoxides using HDMC catalyst.

Run *^a^*	Epoxide	Toluene (mL)	*T*_P_ (°C)	$P_{{\mathrm{CO}}_{2}}$ (MPa)	Catalytic Activity			Polymer Properties		
					Conv. (%)	Yield (%)	$S_{{\mathrm{CO}}_{2}}$ ^*b*^ (%)	*M*_n_ (g mol^−1^)	*Ð*	*DB ^c^*
1 *^d^*	PO	10	105	3	97.5	63.4	40.1	2400	1.83	0.00
2	G	10	110	2	84.3	47.8	22.8	1380	1.72	0.18
3	G	10	120	2	90.3	44.4	32.7	1150	1.49	0.14
4	G	10	130	2	90.7	50.0	21.6	2120	1.90	0.19
5	G	10	140	2	92.4	62.7	18.4	1340	1.50	0.19
6	G	10	120	1	87.2	70.2	18.3	1100	1.45	0.20
7	G	10	120	1.5	90.2	58.4	25.6	1220	1.56	0.17
8	G	10	120	2.5	90.7	33.2	25.9	1670	1.77	0.21
9	G	10	120	3	91.1	29.5	23.1	1400	1.68	0.19
10	G	0	120	2	93.8	62.5	25.8	1030	1.10	0.17
11	G	5	120	2	92.4	47.6	28.1	1080	1.22	0.17
12	G	20	120	2	80.6	64.2	14.2	1120	2.01	0.19

*^a^* Reaction conditions: HDMC catalyst = 20 mg, monomer = 0.3 mol, toluene = 10 mL, *t*_P_ = 6 h. *^b^*  Carbonate content. *^c^* Degree of branching. *^d^* HDMC catalyst = 50 mg, *m*_PPG_ = 1 g, *t*_P_ = 3 h.

## Data Availability

The original contributions presented in this study are included in the article. Further inquiries can be directed to the corresponding authors.

## Supplementary Materials

The following supporting information can be downloaded at: https://www.mdpi.com/article/10.3390/polym17182458/s1, FTIR spectra, XPS profiles, XRD patterns, and TGA curves of DMC catalysts; ^1^H NMR spectra of polyols; supplementary figures and tables.
