# Supplementary material for: Synthesis of Linear and Branched Polycarbonate Polyols via Double Metal Cyanide-Catalyzed Ring-Opening (Co)polymerization of Epoxides
Source: Polymers (Basel). 2025 Sep 11;17(18):2458. doi: 10.3390/polym17182458 (PMC12473593; doi:10.3390/polym17182458)
Supplement: Supplementary file 1 [file polymers-17-02458-s001.zip › polymers-3852569-supplementary.pdf]

Supporting Information

Synthesis of Linear and Branched Polycarbonate Polyols via Double Metal Cyanide-Catalyzed Ring-Opening (Co)polymerization of Epoxides

Won Seok Jae [1], Ha-Kyung Choi [1], Han Su Lee [1], Chinh Hoang Tran [1,2,*], Chi Le Hoang Tran [3], Khoa Anh Trinh [3] and Il Kim [1,*]

[1] School of Chemical Engineering, Pusan National University, Busandaehag-ro 63-2, Geumjeong-gu, Busan 46241, Republic of Korea; coly2343@pusan.ac.kr (W.S.J.); aomghwm0116@pusan.ac.kr (H.-K.C.); tyghbn22@pusan.ac.kr (H.S.L.)

[2] Institute of Advanced Technology, Vietnam Academy of Science and Technology, TL29 Street, An Phu Dong, Ho Chi Minh 70000, Vietnam

[3] FPT University, FPT Polytechnic, Nguyen Van Cu Street, An Binh, Can Tho 90000, Vietnam; chithl@fpt.edu.vn (C.L.H.T.); khoata17@fe.edu.vn (K.A.T.)

* Correspondence: chinhtran@pusan.ac.kr (C.H.T.); ilkim@pusan.ac.kr (I.K.); Tel.: +82-10-6399-5903 (I.K.)

Table of Contents

1. Supplementary figures....4

1.1 Characterization of DMC catalysts....4

Figure S1 FTIR spectra of the DMC-DEP prepared using various temperature and DMC-H_3PO_3....4

Figure S2 FTIR spectra of the DMC-$P(OEt)_3$ prepared at various temperatures and without co-CA....4

Figure S3 FTIR spectra of the DMC-TEP prepared using various temperature. 5

Figure S4 FTIR spectra of the DMC-TEP prepared using various amounts of CA. 5

Figure S5 FTIR spectra of the DMC-*t*BuOH prepared using various amounts of CA....6

Figure S6 XRD patterns of the prepared DMC catalysts. 6

Figure S7 XRD patterns of the DMC-DEP and $P(OEt)_3$ prepared at various temperature. (◯) denote the monoclinic (*P*11*m*) phases....7

Figure S8 XRD patterns of the DMC-TEP and DMC-DEP prepared using various amounts of CAs. (□), and (◯) denote the cubic (*Fm*-3*m*), and monoclinic (*P*11*m*) phases, respectively. 7

Figure S9 SEM images of the DMC-pure and the optimized DMC-DEP catalysts. 8

Figure S10 TGA curve of the prepared DMC catalysts 9

1.2 Catalytic reaction 6

Figure S11 Reaction rate curves of the ROP of PO obtained by DMC-$P(OEt)_3$ prepared using various amount of CA and catalyst preparation temperature. Reaction condition: Catalyst loading (n_{Zn}) = 0.3 mmol, PO = 3.5 mol, PPG-600 = 50 mmol, T_P = 115 °C. 6

Figure S12 Reaction rate curves of the ROP of PO obtained by DMC-DEP prepared using various amount of CA and catalyst preparation temperature. Reaction condition: Catalyst loading (n_{Zn}) = 0.3 mmol, PO = 3.5 mol, PPG-600 = 50 mmol, T_P = 115 °C. 6

Figure S13 Reaction rate curves of the ROP of PO obtained by DMC-TEP prepared using various amount of CA and catalyst preparation temperature. Reaction condition: Catalyst loading (n_{Zn}) = 0.3 mmol, PO = 3.5 mol, PPG-600 = 50 mmol, T_P = 115 °C. 7

Figure S14 Reaction rate curves of the ROP of PO obtained by DMC-TEP prepared using various co-CAs. Reaction condition: Catalyst loading (n_{Zn}) = 0.3 mmol, PO = 3.5 mol, PPG-400 = 50 mmol, T_P = 115 °C. 7

Figure S15 Reaction rate curves of the ROP of PO obtained by DMC-*t*BuOH prepared using various amount of CA. Reaction condition: Catalyst loading (n_{Zn}) = 0.3 mmol, PO = 3.5 mol, PPG-400 = 50 mmol, T_P = 115 °C. 8

Figure S16 ^{1}H NMR spectrum (400 MHz, $CDCl_3$) of the PPG produced by DMC-DMP. Polymerization condition: Catalyst amount = 100 mg, PO = 200 mol, PPG-600 = 50 mmol, T_P = 115 °C. 9

Figure S17 ^{1}H NMR spectrum (400 MHz, $CDCl_3$) of the PPG produced by DMC-DEP. Polymerization Reaction condition: Catalyst amount = 100 mg, PO = 200 mol, PPG-600 = 50 mmol, T_P = 115 °C....10

Figure S18 ^{1}H NMR spectrum (400 MHz, $CDCl_3$) of the PPG produced by DMC-D*t*BuP. Polymerization Reaction condition: Catalyst amount = 100 mg, PO = 200 mol, PPG-600 = 50 mmol, T_P = 115 °C....11

Figure S19 ^{1}H NMR spectrum (400 MHz, $CDCl_3$) of the PPG produced by DMC-$P(OMe)_3$. Polymerization Reaction condition: Catalyst amount = 100 mg, PO = 200 mol, PPG-600 = 50 mmol, T_P = 115 °C....12

Figure S20 ^{1}H NMR spectrum (400 MHz, $CDCl_3$) of the PPG produced by DMC-$P(OEt)_3$. Polymerization Reaction condition: Catalyst amount = 100 mg, PO = 200 mol, PPG-600 = 50 mmol, T_P = 115 °C....13

Figure S21 ^{1}H NMR spectrum (400 MHz, $CDCl_3$) of the PPG produced by DMC-TEP. Polymerization Reaction condition: Catalyst amount = 100 mg, PO = 200 mol, PPG-600 = 50 mmol, T_P = 115 °C....20

2. Supplementary tables 221

Table S1. DMC catalysts prepared using $ZnCl_2$(15 mmol), $K_3Co(CN)_6$ (1.5 mmol), and various type of OPC CAs. 221

3. References....22

1. Supplementary figures

1.1 Characterization of DMC catalysts

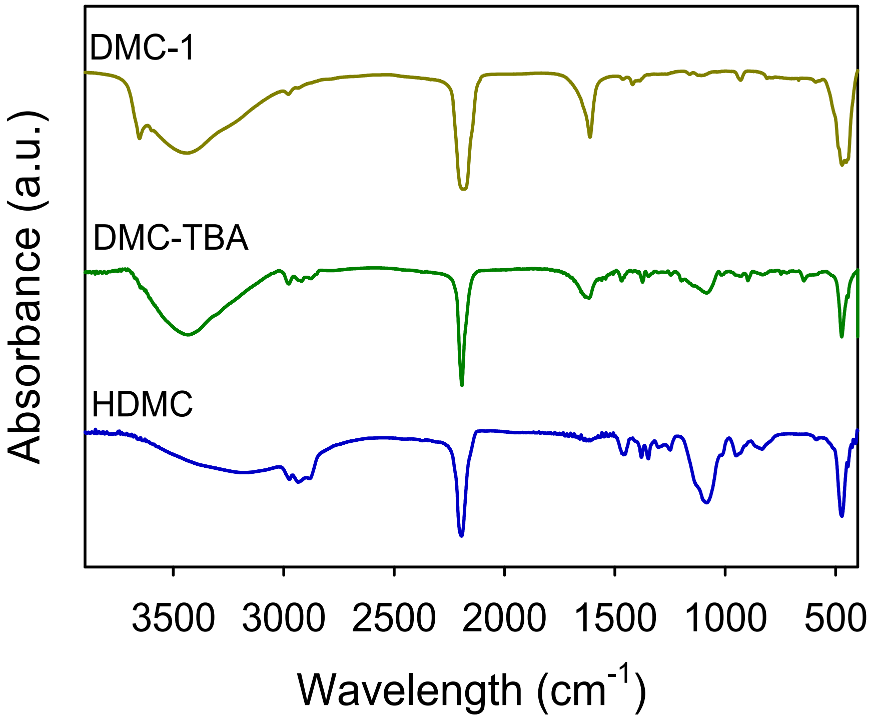

Figure S1 FTIR spectra of the DMC catalysts.

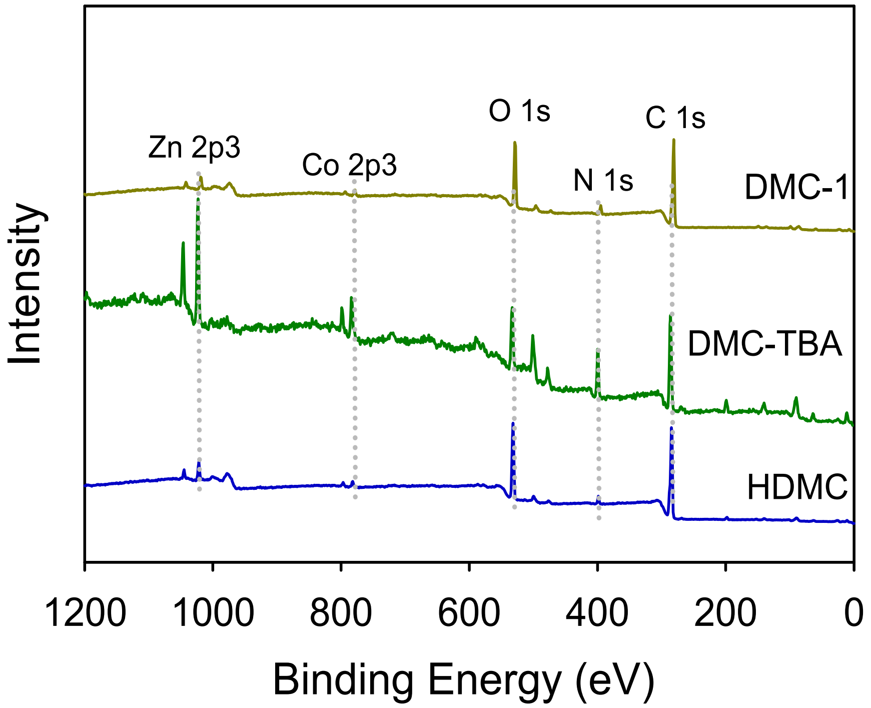

Figure S2 XPS spectra of the DMC catalysts.

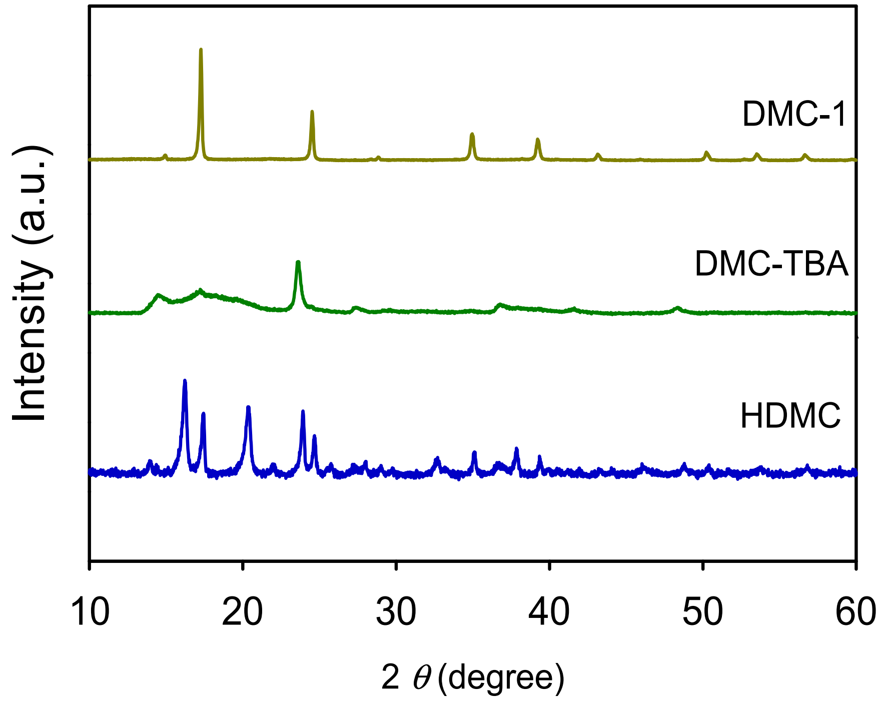

Figure S3 XRD patterns of the DMC catalysts.

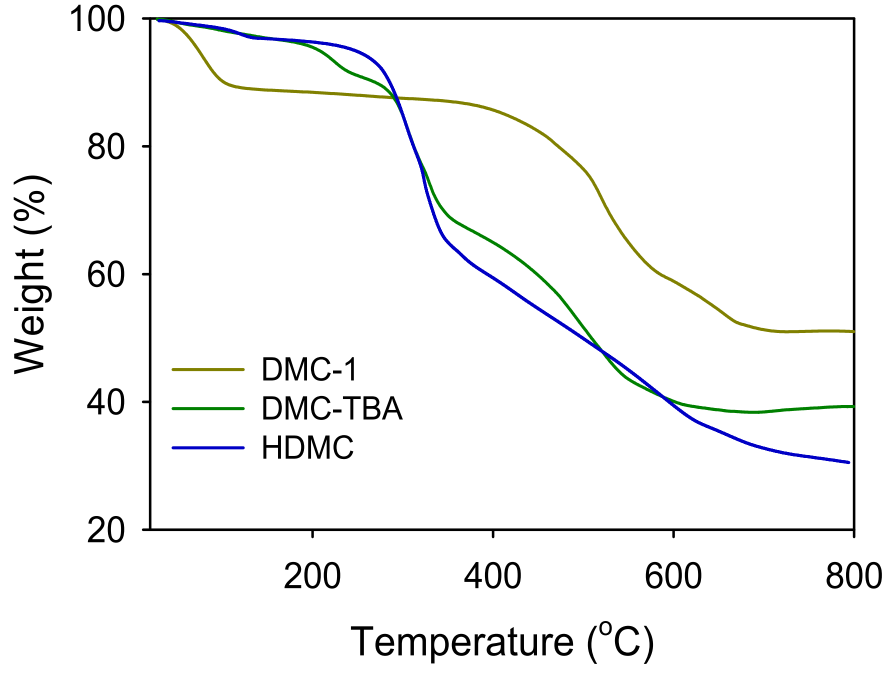

Figure S4 TGA curve of the DMC catalysts.

1.2 ROP of epoxide

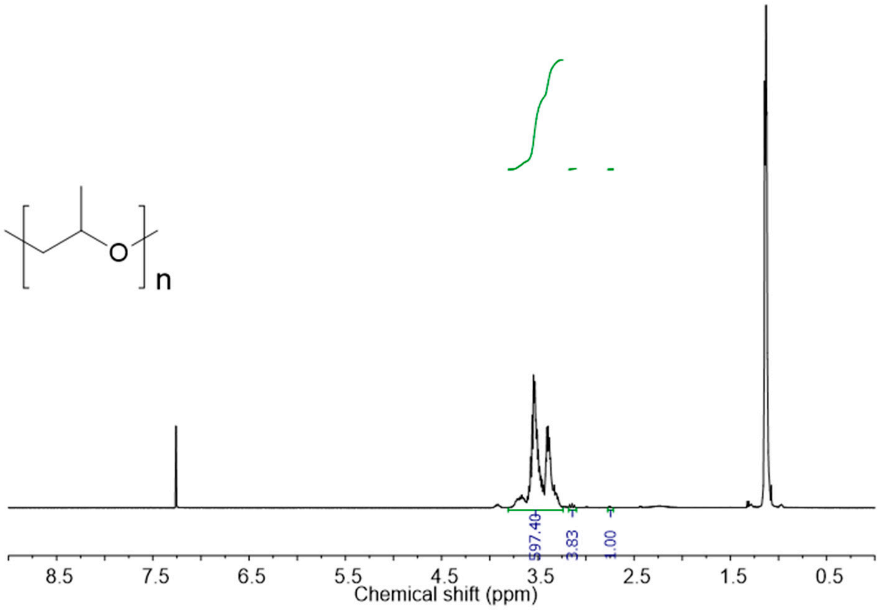

Figure S5 ^{1}H NMR (400 MHz, $CDCl_3$) spectrum of the poly(propylene oxide) polyol produced by HDMC via batch ROP of PO. Reaction condition: HDMC catalyst = 2 mg, $m_{\text{PPG initiator}}$ = 0.2 g, PO = 30 mmol, T_P = 115 °C.

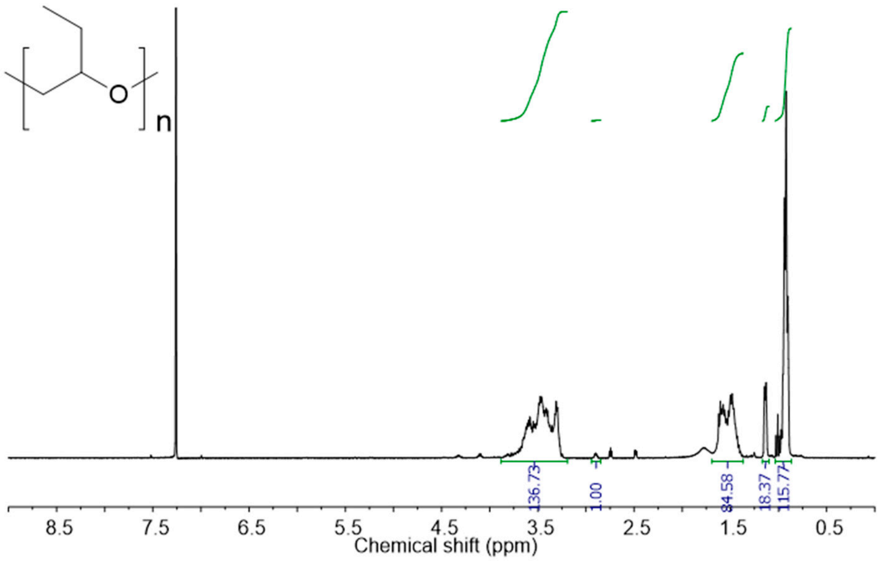

Figure S6 ^{1}H NMR (400 MHz, $CDCl_3$) spectrum of the poly(epoxybutane) polyol produced by HDMC via batch ROP of EB. Reaction condition: HDMC catalyst = 2 mg, $m_{\text{PPG initiator}}$ = 0.2 g, EB = 30 mmol, T_P = 115 °C.

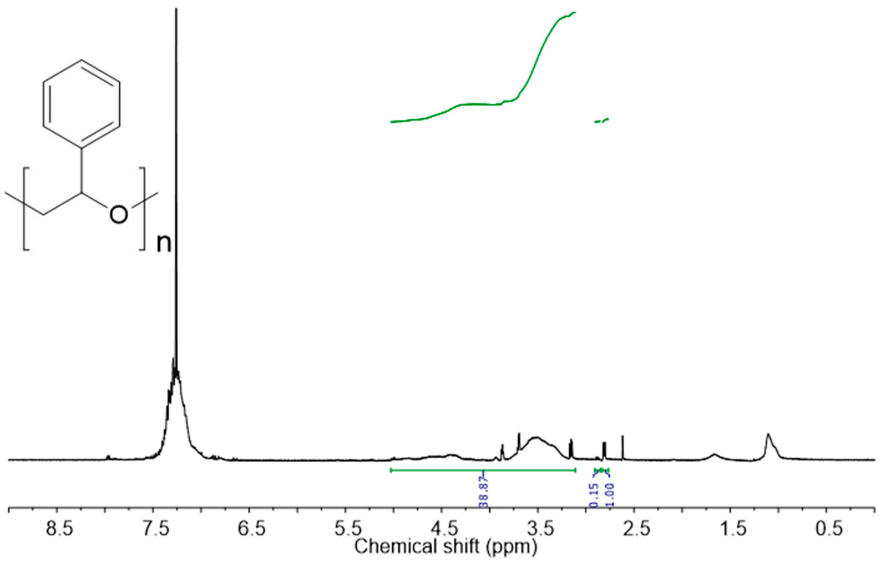

Figure S7 ^{1}H NMR (400 MHz, $CDCl_3$) spectrum of the poly(styrene oxide) polyol produced by HDMC via batch ROP of SO. Reaction condition: HDMC catalyst = 2 mg, $m_{PPG\ initiator}$ = 0.2 g, SO = 30 mmol, T_P = 115 °C.

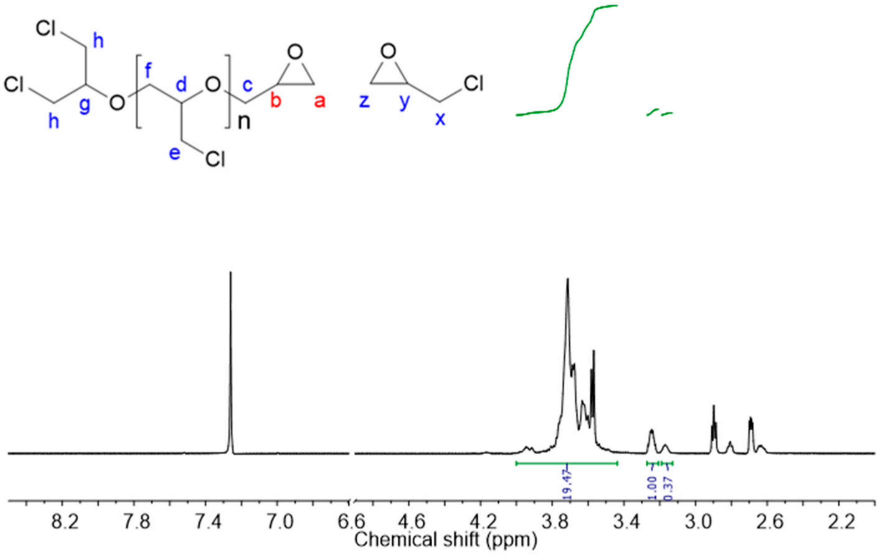

Figure S8 ^{1}H NMR (400 MHz, $CDCl_3$) spectrum of the poly(epichlorohydrin) polyol produced by HDMC via batch ROP of ECH. Reaction condition: HDMC catalyst = 2 mg, ECH = 30 mmol, T_P = 115 °C.

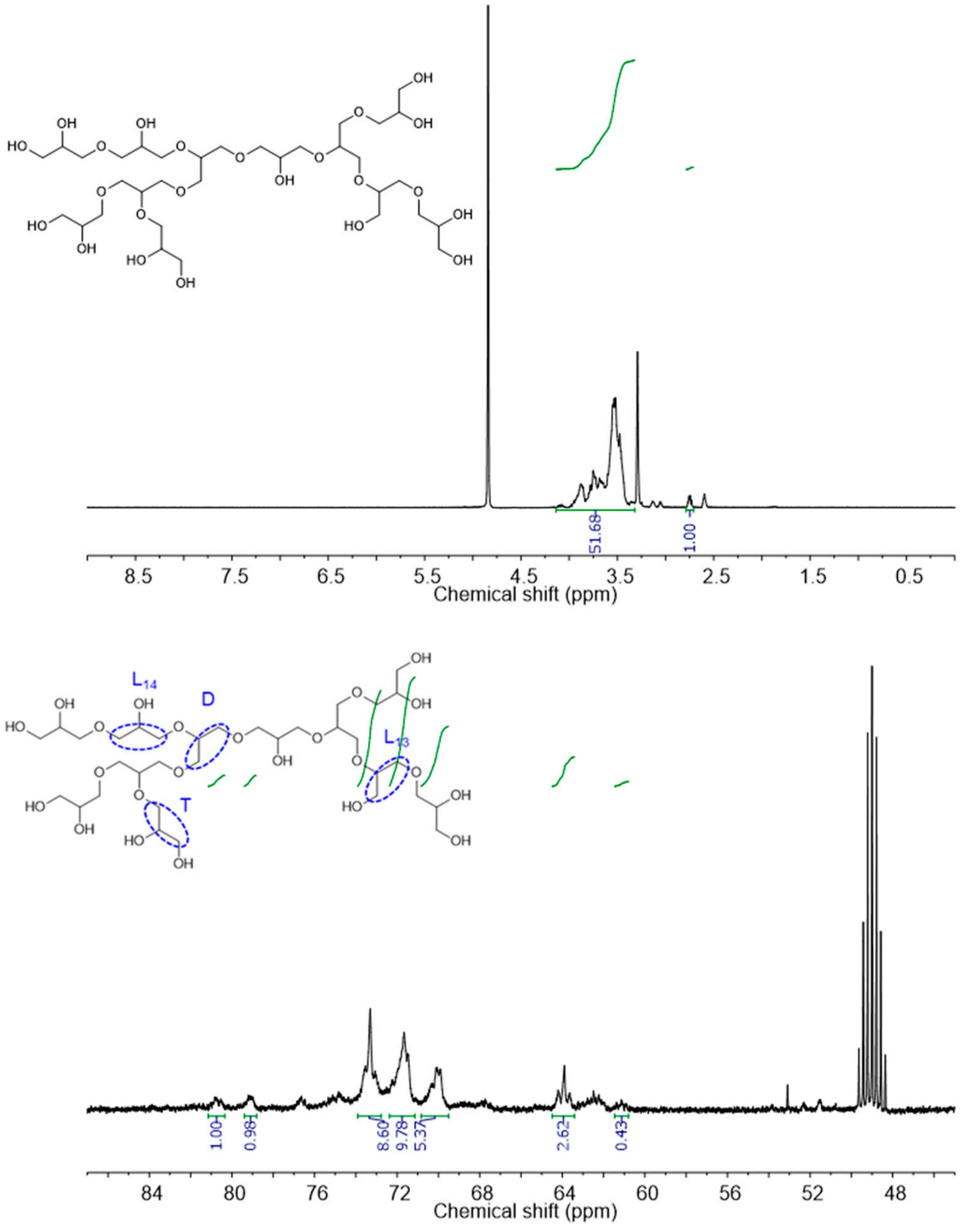

Figure S9 ^{1}H NMR and ^{13}C NMR (400 MHz, CD_3OD) spectra of the polyglycidol produced by HDMC via batch ROP of glycidol. Reaction condition: HDMC catalyst = 2 mg, glycidol = 0.03 mol, T_P = 115 °C.

1.3 Copolymerization of CO_2 with epoxide

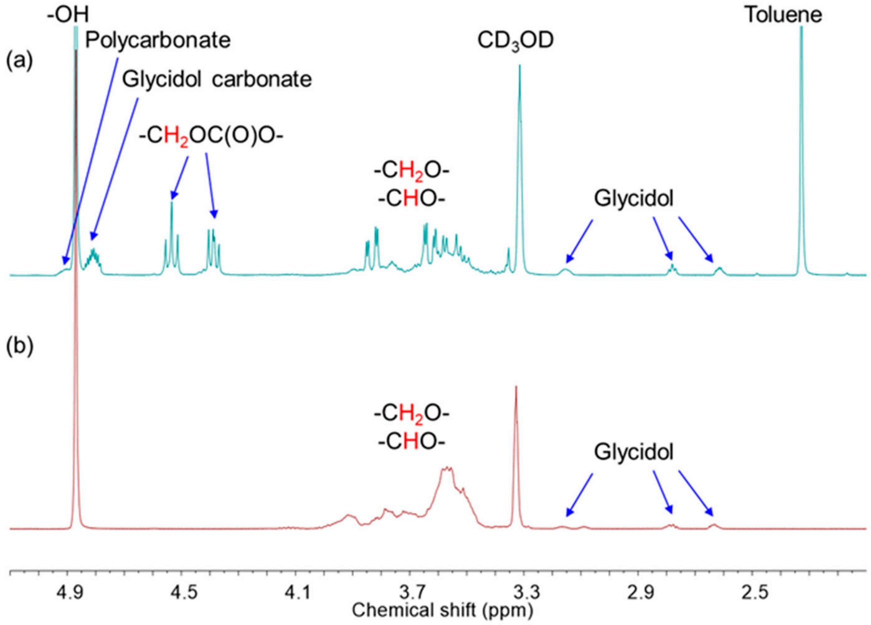

Figure S10 ^{1}H NMR (400 MHz, CD_3OD) spectra of the crude reaction mixture of the copolymerization of CO_2 with glycidol (a) and ROMBP of glycidol (b) using HDMC catalyst.

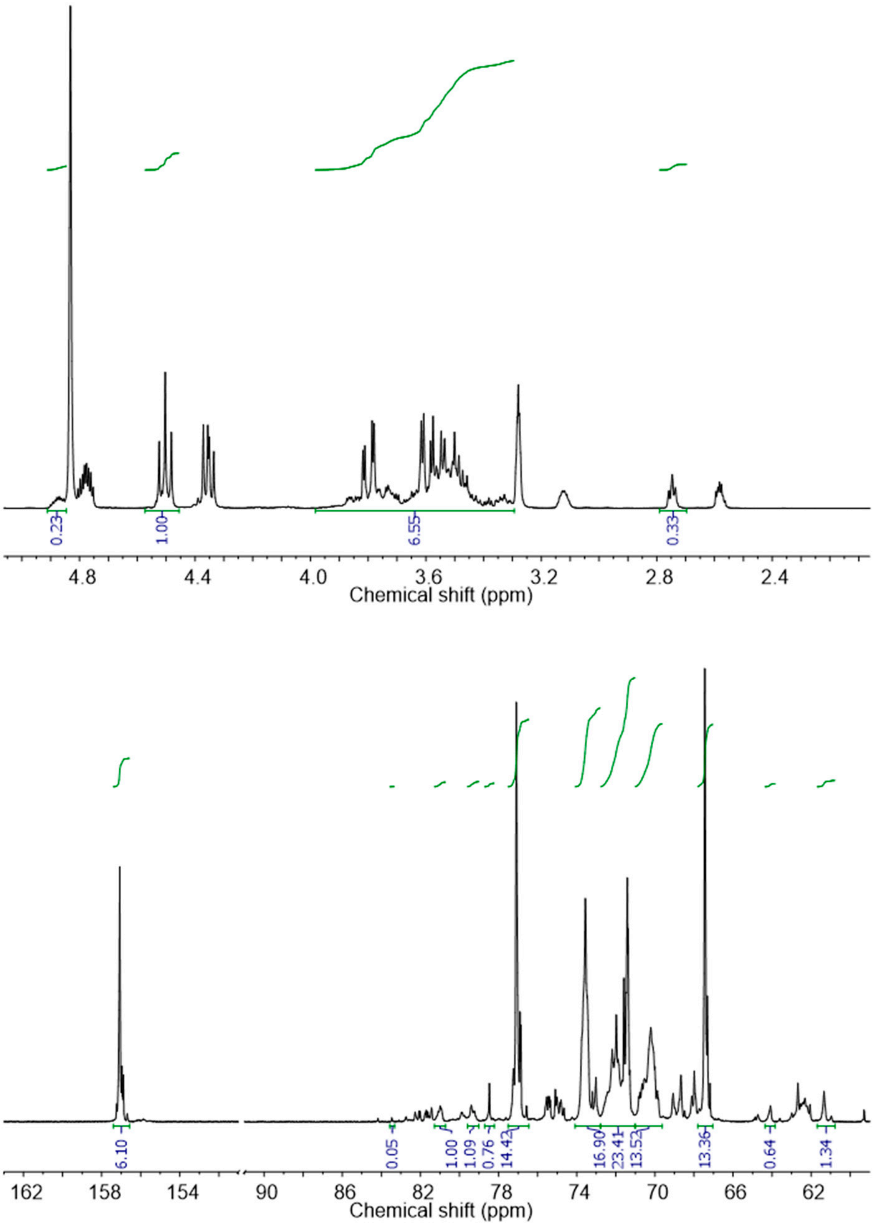

Figure S11 ^{1}H NMR and ^{13}C NMR (400 MHz, CD_3OD) spectra of crude reaction mixture of the copolymerization of CO_2 with glycidol using HDMC catalyst. Reaction condition: HDMC catalyst = 20 mg, glycidol = 0.3 mol, toluene = 10 mL, P_{CO_2} = 2 MPa, T_P = 110 °C, t_P = 6 h.

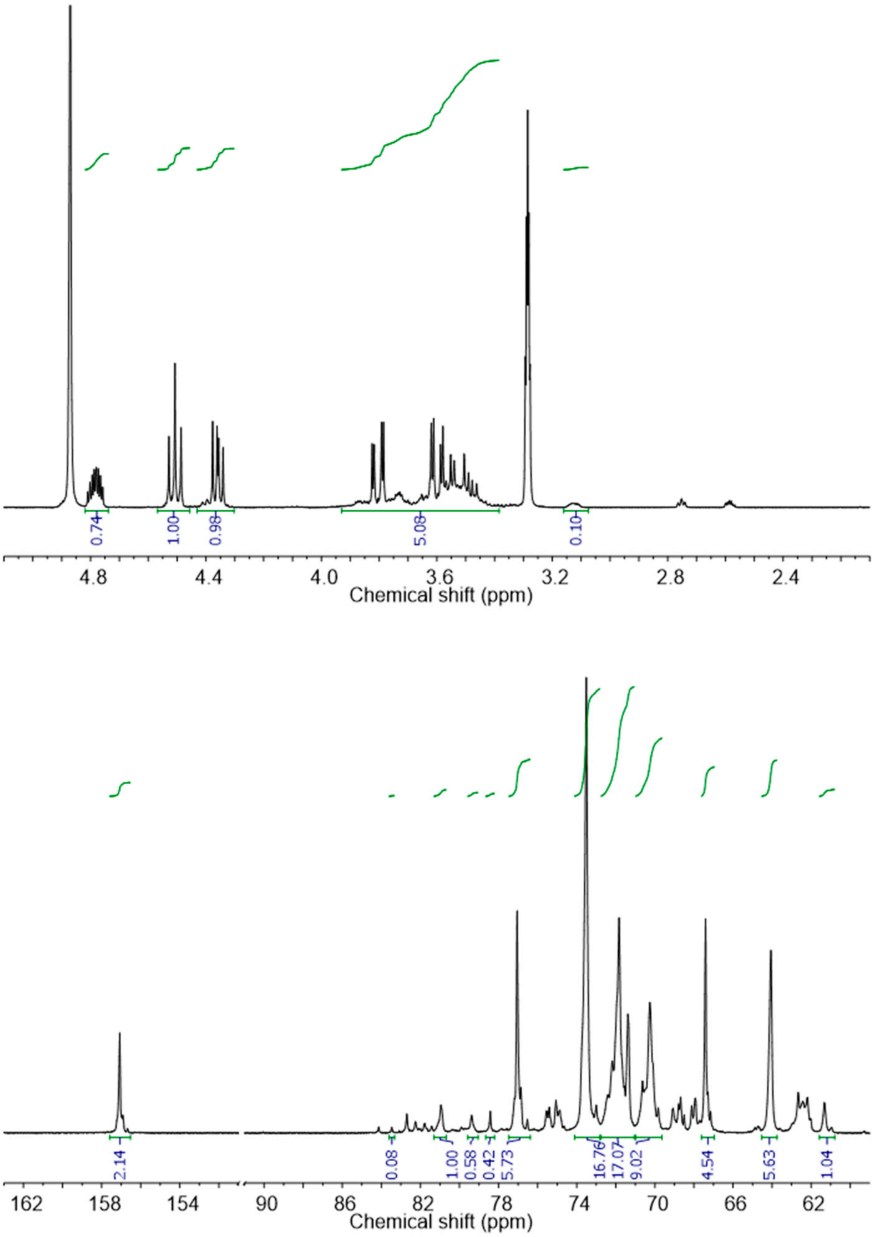

Figure S12 ^{1}H NMR and ^{13}C NMR (400 MHz, CD_3OD) spectra of crude reaction mixture of the copolymerization of CO_2 with glycidol using HDMC catalyst. Reaction condition: HDMC catalyst = 20 mg, glycidol = 0.3 mol, toluene = 10 mL, P_{CO_2} = 2 MPa, T_P = 120 °C, t_P = 6 h.

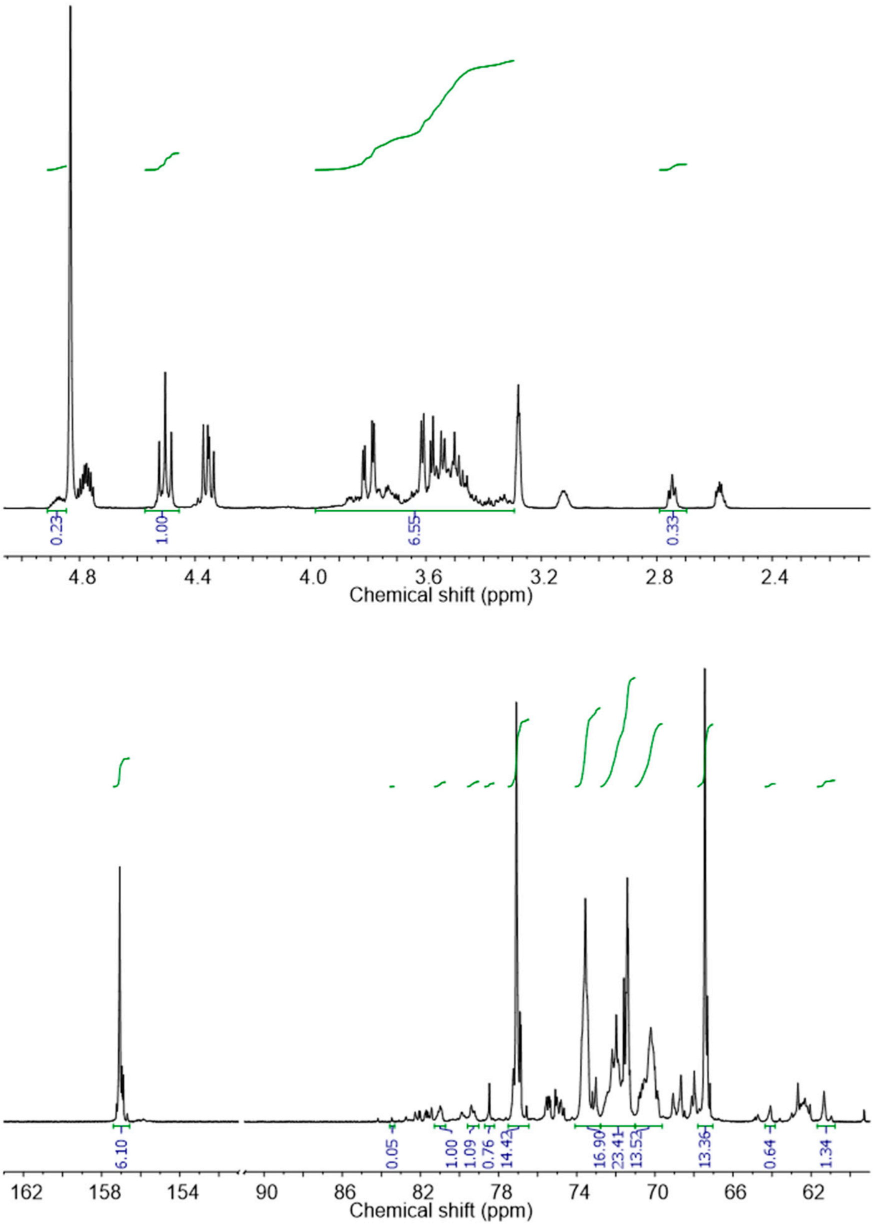

Figure S13 ^{1}H NMR and ^{13}C NMR (400 MHz, CD_3OD) spectra of crude reaction mixture of the copolymerization of CO_2 with glycidol using HDMC catalyst. Reaction condition: HDMC catalyst = 20 mg, glycidol = 0.3 mol, toluene = 10 mL, P_{CO_2} = 2 MPa, T_P = 130 °C, t_P = 6 h.

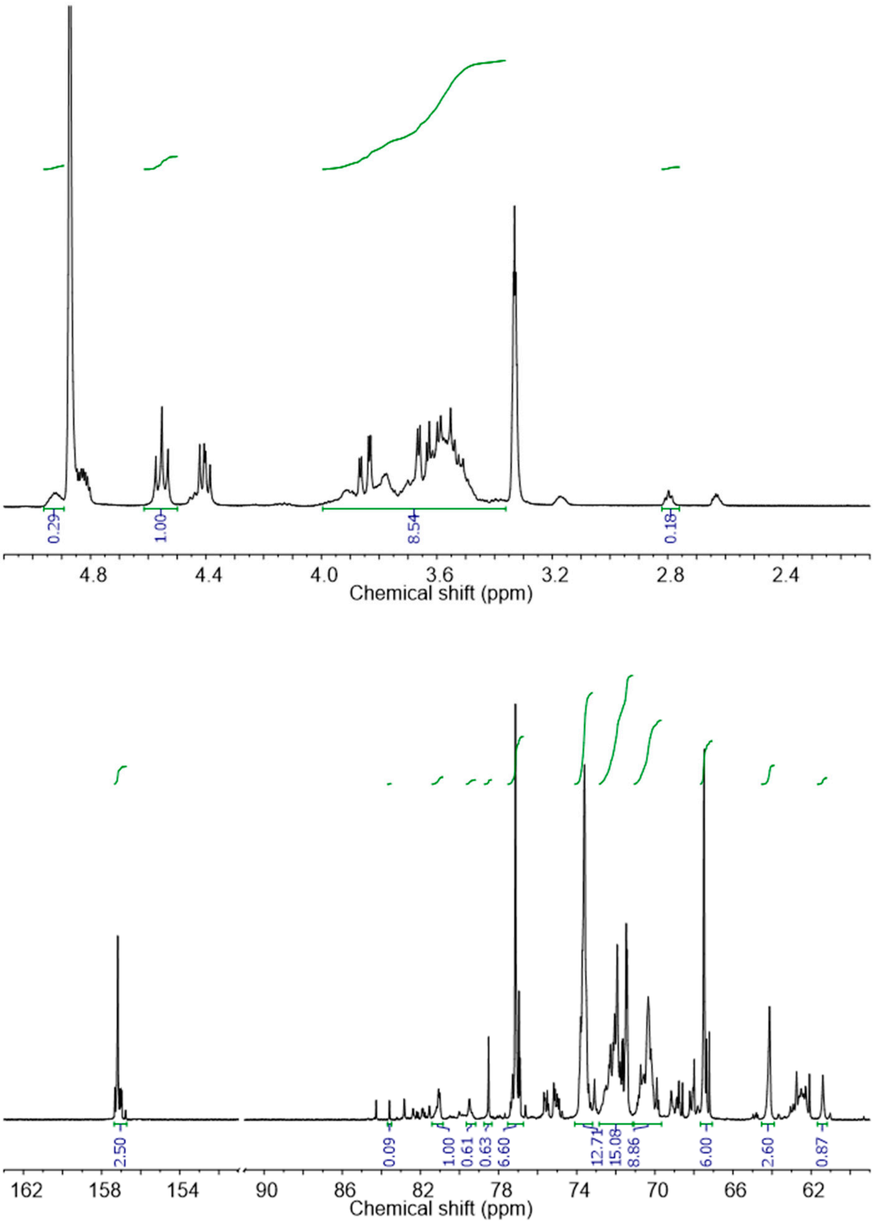

Figure S14 ^{1}H NMR and ^{13}C NMR (400 MHz, CD_3OD) spectra of crude reaction mixture of the copolymerization of CO_2 with glycidol using HDMC catalyst. Reaction condition: HDMC catalyst = 20 mg, glycidol = 0.3 mol, toluene = 10 mL, P_{CO_2} = 2 MPa, T_P = 140 °C, t_P = 6 h.

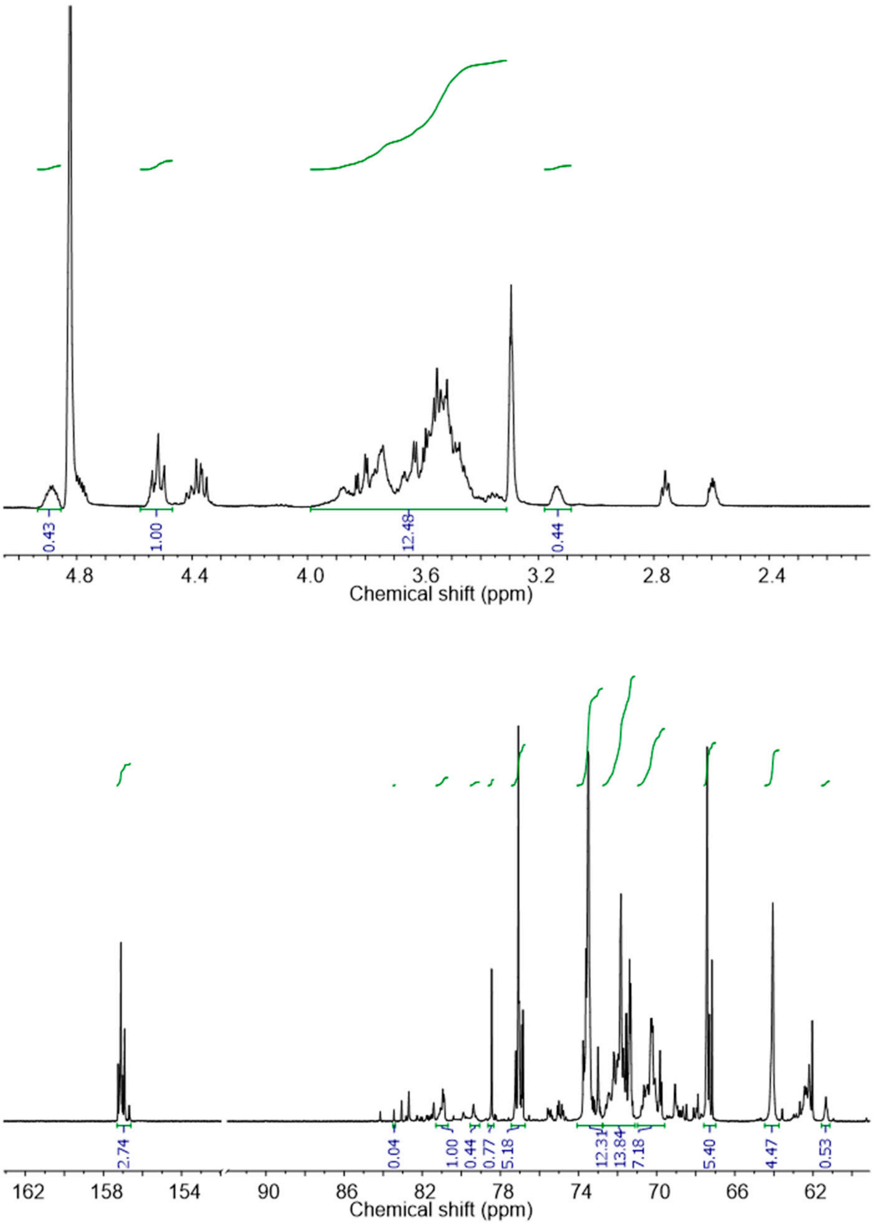

Figure S15 ^{1}H NMR and ^{13}C NMR (400 MHz, CD_3OD) spectra of crude reaction mixture of the copolymerization of CO_2 with glycidol using HDMC catalyst. Reaction condition: HDMC catalyst = 20 mg, glycidol = 0.3 mol, toluene = 10 mL, P_{CO_2} = 1 MPa, T_P = 120 °C, t_P = 6 h.

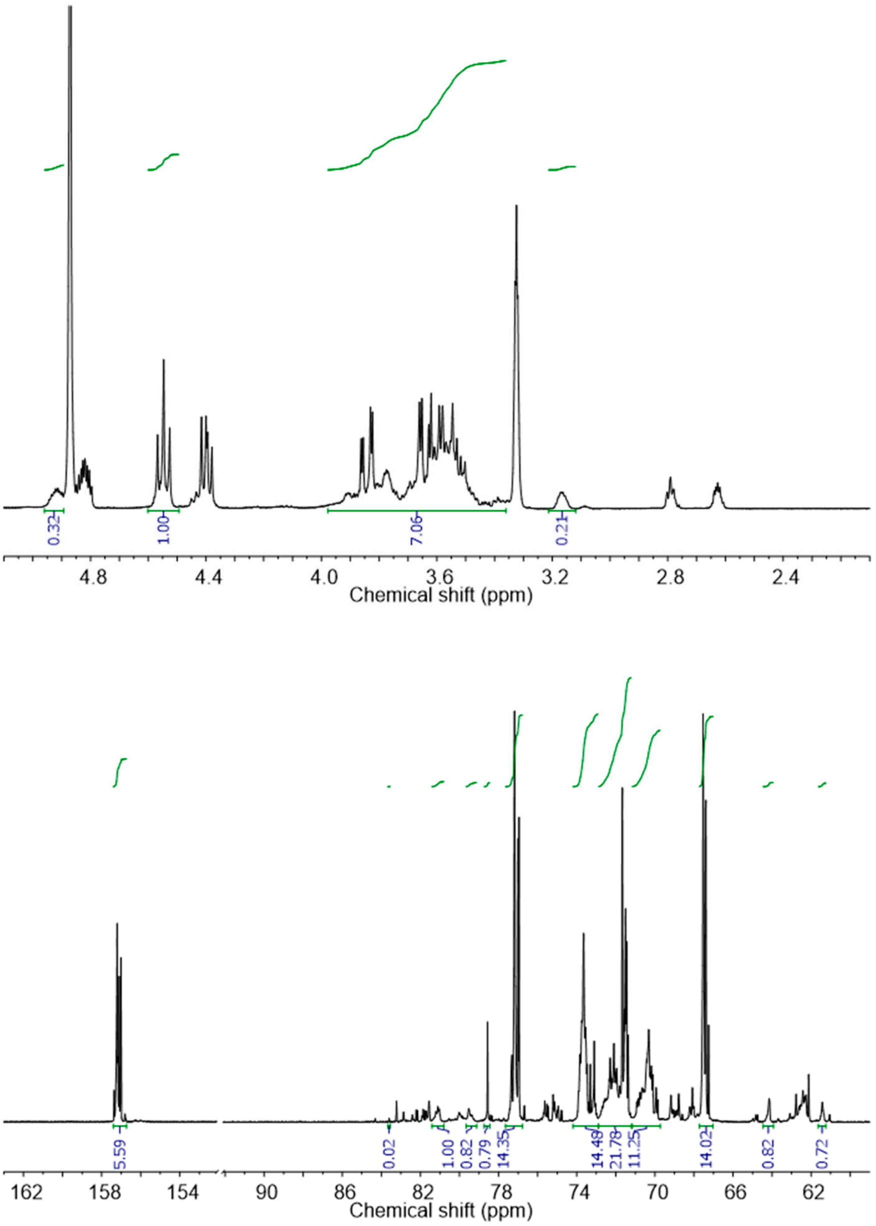

Figure S16 ^{1}H NMR and ^{13}C NMR (400 MHz, CD_3OD) spectra of crude reaction mixture of the copolymerization of CO_2 with glycidol using HDMC catalyst. Reaction condition: HDMC catalyst = 20 mg, glycidol = 0.3 mol, toluene = 10 mL, P_{CO_2} = 1.5 MPa, T_P = 120 °C, t_P = 6 h.

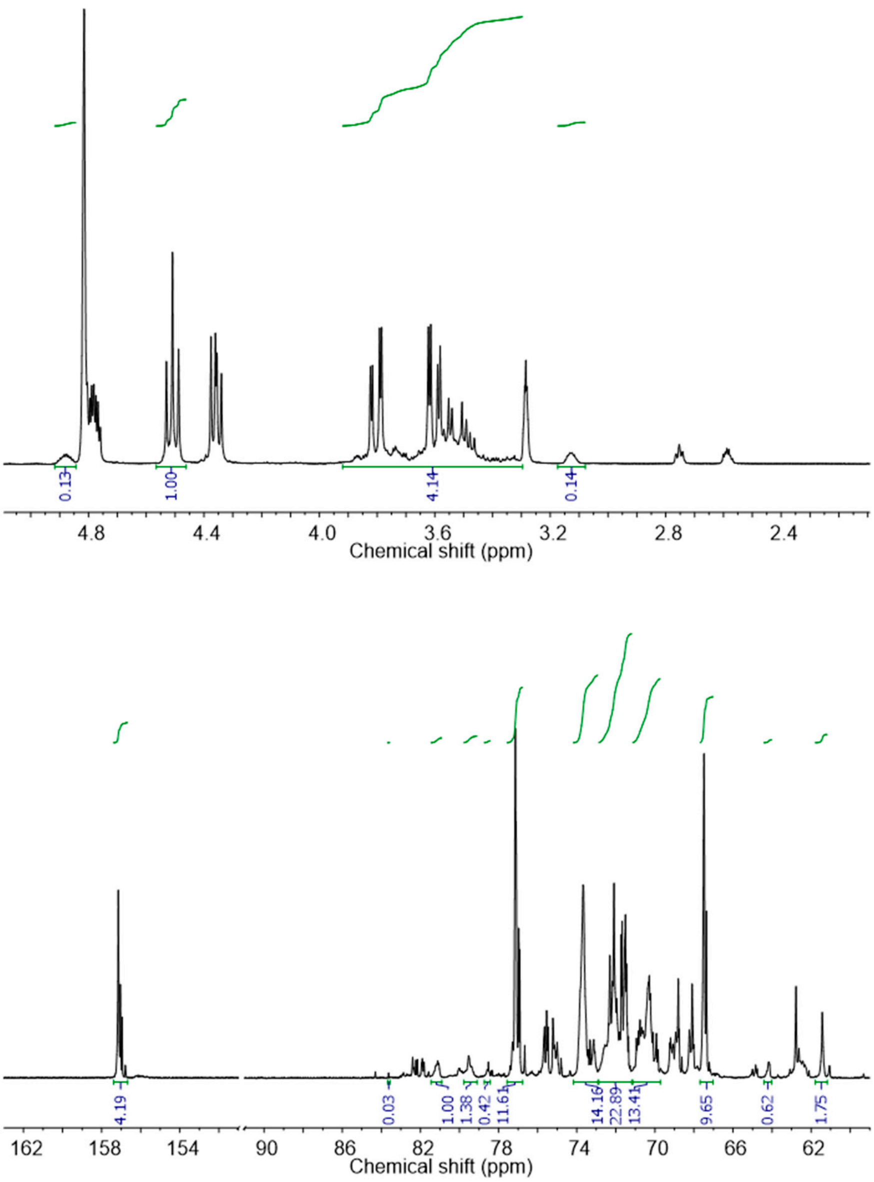

Figure S17 ^{1}H NMR and ^{13}C NMR (400 MHz, CD_3OD) spectra of crude reaction mixture of the copolymerization of CO_2 with glycidol using HDMC catalyst. Reaction condition: HDMC catalyst = 20 mg, glycidol = 0.3 mol, toluene = 10 mL, P_{CO_2} = 2.5 MPa, T_P = 120 °C, t_P = 6 h.

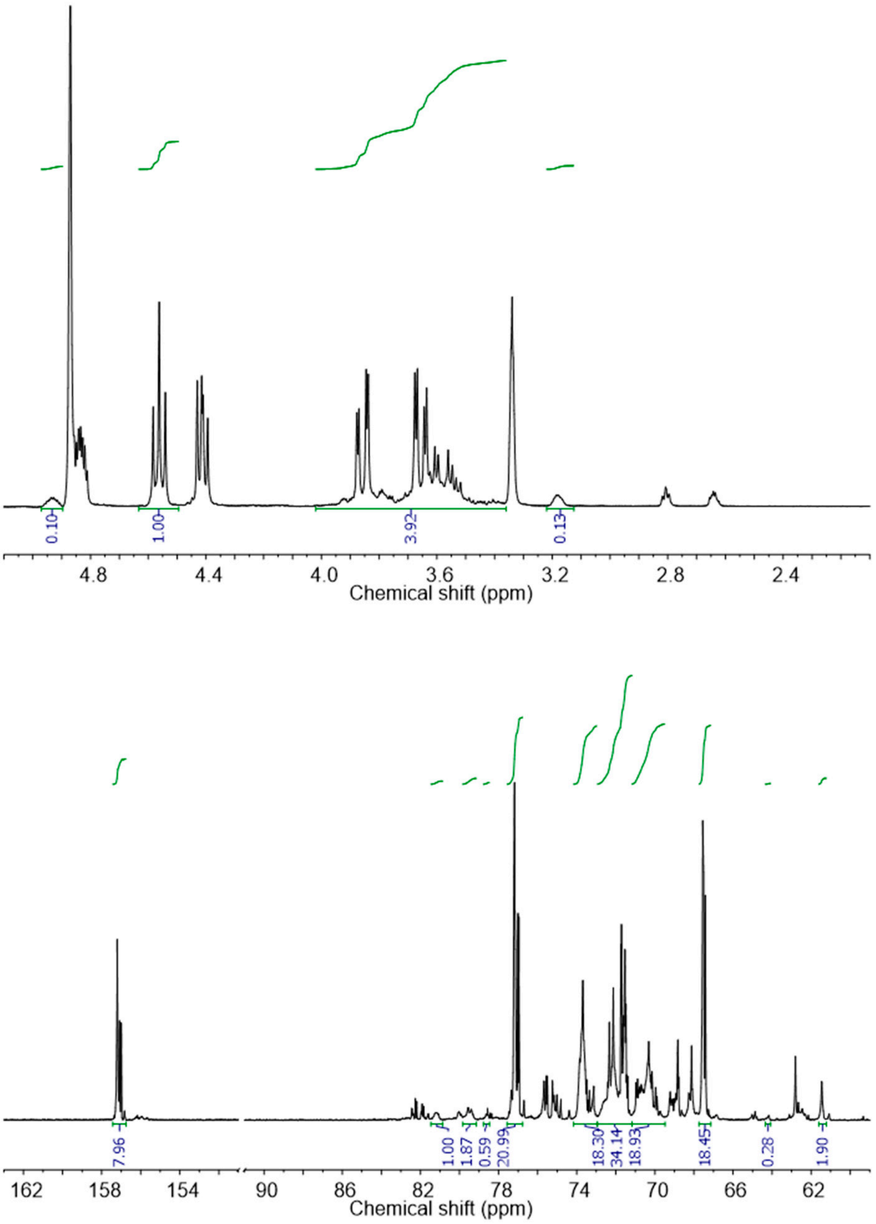

Figure S18 ^{1}H NMR and ^{13}C NMR (400 MHz, CD_3OD) spectra of crude reaction mixture of the copolymerization of CO_2 with glycidol using HDMC catalyst. Reaction condition: HDMC catalyst = 20 mg, glycidol = 0.3 mol, toluene = 10 mL, P_{CO_2} = 3 MPa, T_P = 120 °C, t_P = 6 h.

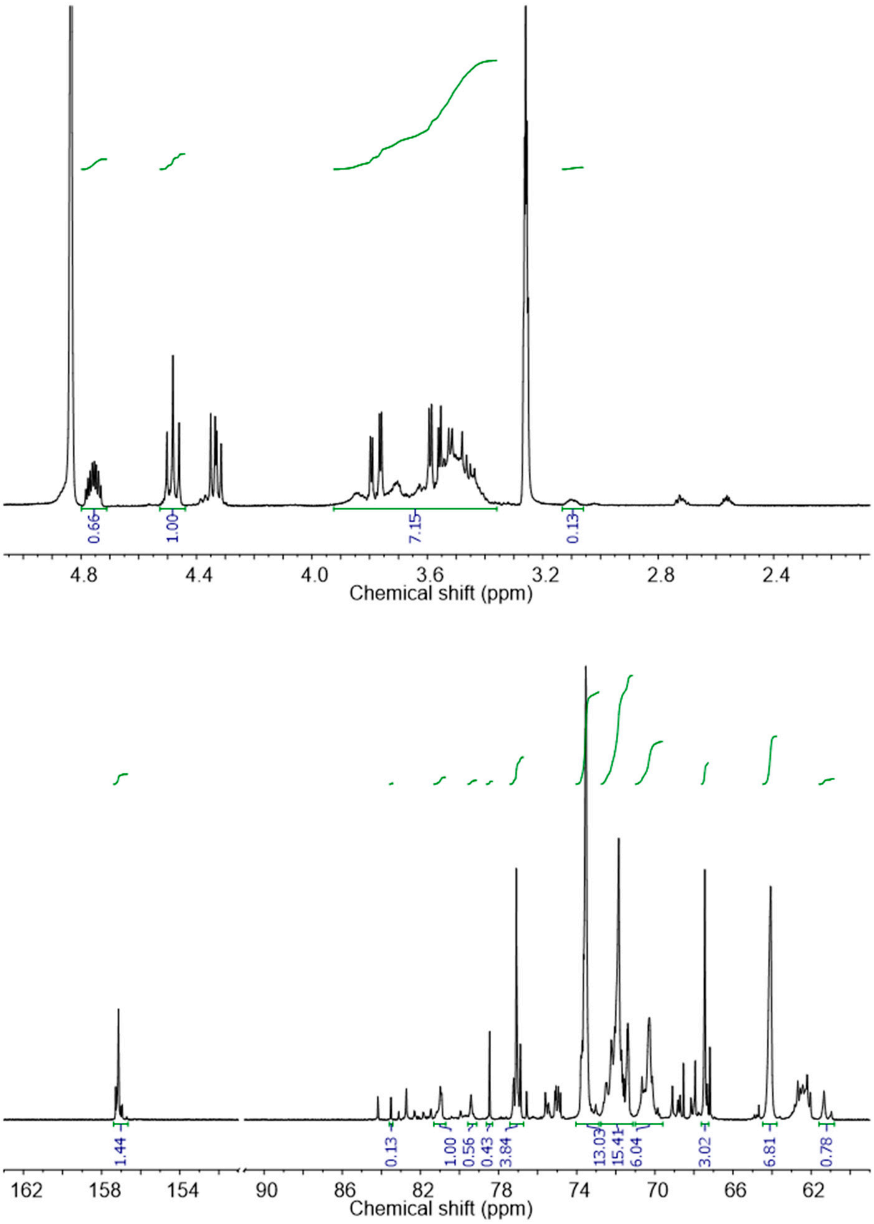

Figure S19 ^{1}H NMR and ^{13}C NMR (400 MHz, CD_3OD) spectra of crude reaction mixture of the copolymerization of CO_2 with glycidol using HDMC catalyst. Reaction condition: HDMC catalyst = 20 mg, glycidol = 0.3 mol, toluene = 0 mL, P_{CO_2} = 2 MPa, T_P = 120 °C, t_P = 6 h.

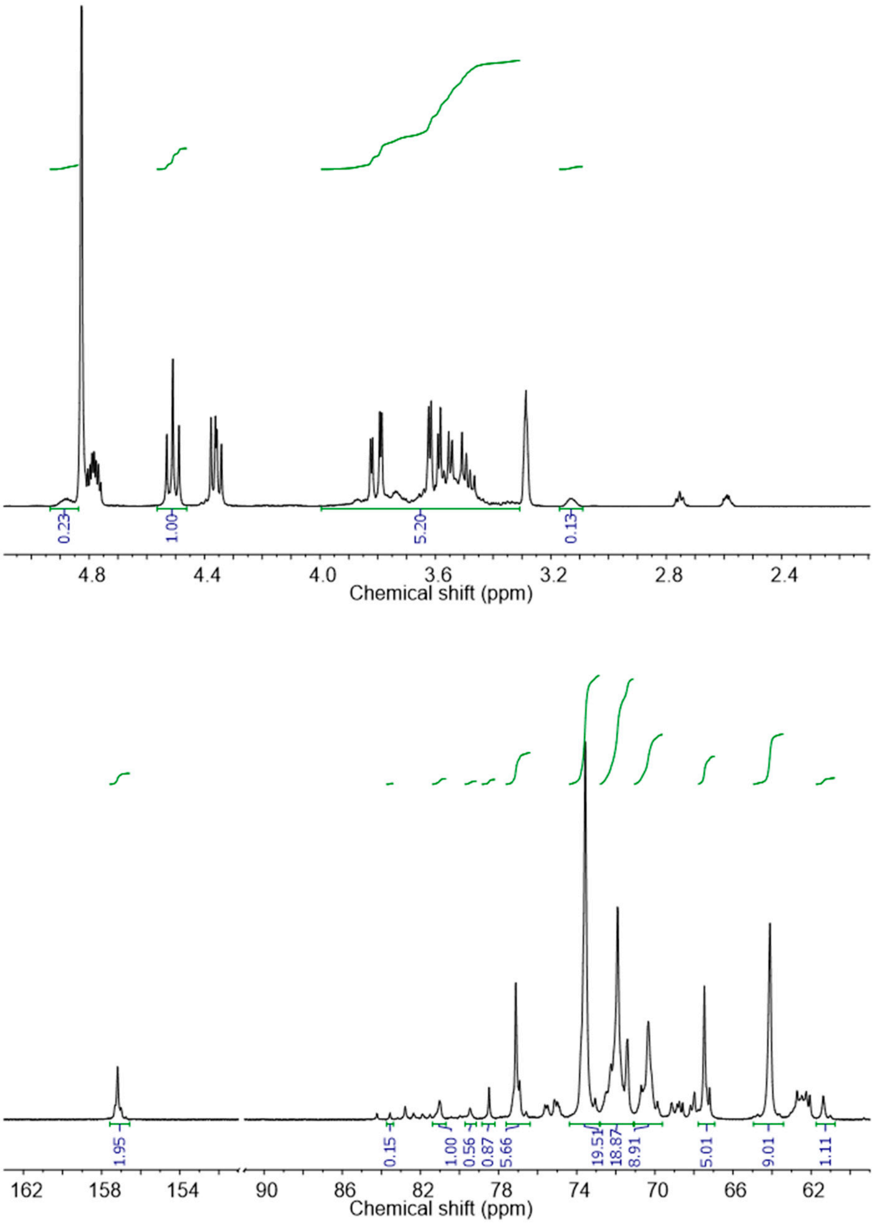

Figure S20 ^{1}H NMR and ^{13}C NMR (400 MHz, CD_3OD) spectra of crude reaction mixture of the copolymerization of CO_2 with glycidol using HDMC catalyst. Reaction condition: HDMC catalyst = 20 mg, glycidol = 0.3 mol, toluene = 5 mL, P_{CO_2} = 2 MPa, T_P = 120 °C, t_P = 6 h.

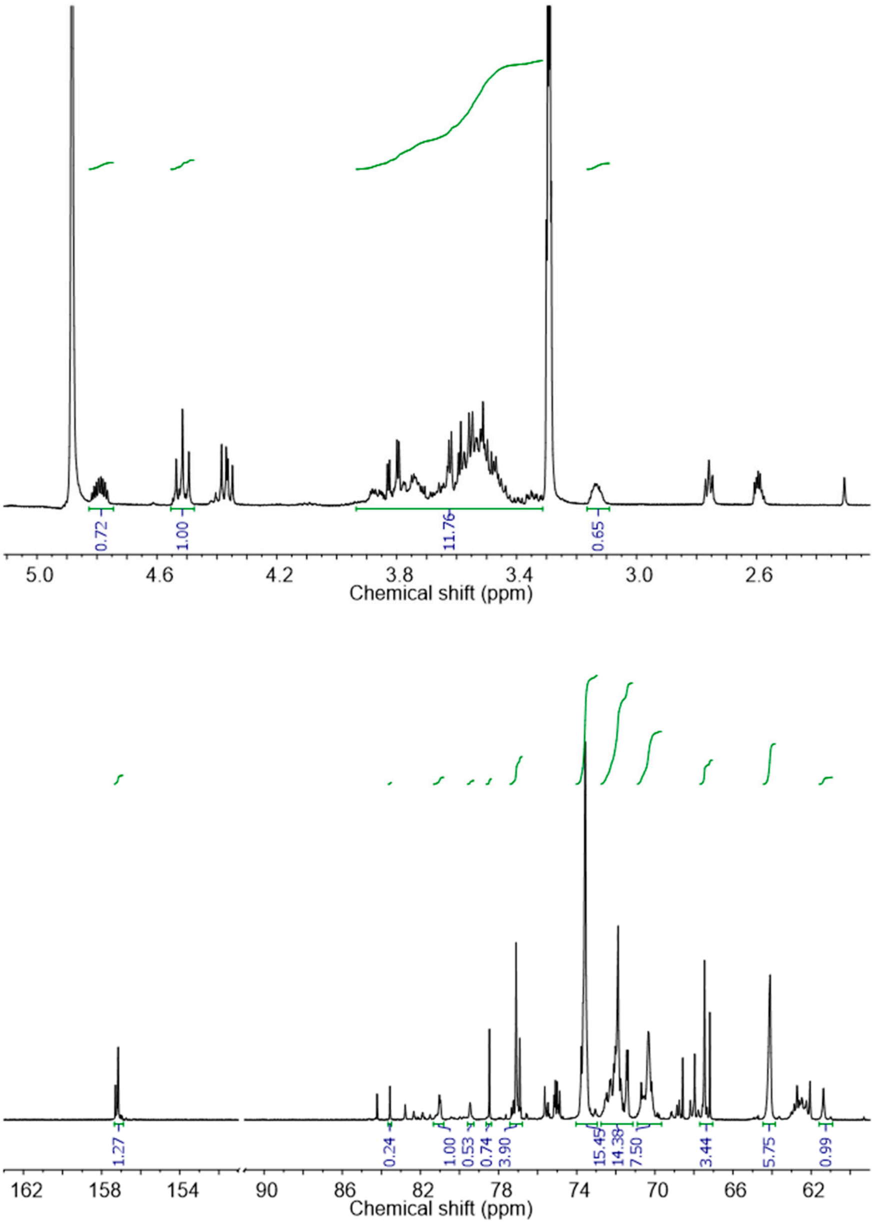

Figure S21 ^{1}H NMR and ^{13}C NMR (400 MHz, CD_3OD) spectra of crude reaction mixture of the copolymerization of CO_2 with glycidol using HDMC catalyst. Reaction condition: HDMC catalyst = 20 mg, glycidol = 0.3 mol, toluene = 20 mL, P_{CO_2} = 2 MPa, T_P = 120 °C, t_P = 6 h.

2. Supplementary tables

Table S1. Elemental analysis results for the DMC catalysts

Catalyst	ICP-OES (wt.%)		Elemental analysis (wt.%)			TGA (wt.%)			Estimated catalyst formulation
	Zn	Co	C	H	N	CA	Pluronic (PL)	H_2O	
DMC-1	24.2	15.6	21.3	2.1	20.9	–	–	10.1	$Zn_{1.40}Co(CN)_{5.64}\cdot 2.13H_2O\cdot 1.21Cl^-$
HDMC	17.9	9.4	30.3	3.7	14.6	–	35.8	3.2	$Zn_{1.71}Co(CN)_{6.53}\cdot 0.12PL\cdot 1.10H_2O\cdot 1.13Cl^-$
DMC-TBA[a]	23.9	10.7	29.3	3.3	16.3	7.2	24.1	1.9	$Zn_{2.01}Co(CN)_{6.41}\cdot 0.53CA\cdot 0.02PL\cdot 0.58H_2O\cdot 0.29Cl^-$

[a] Obtained from ref. [56].

3. References

[56] Tran, C. H.; Pham, L. T. T.; Lee, Y.; Jang, H. B.; Kim, S.; Kim, I., Mechanistic Insights on Zn(II)−Co(III) Double Metal Cyanide-Catalyzed Ring-Opening Polymerization of Epoxides. *J. Catal.* **2019,** *372*, 86−102.
